# Unraveling the mystery of canopy dieback caused by citrus disease Huanglongbing and its link to hypoxia stress

**DOI:** 10.3389/fpls.2023.1119530

**Published:** 2023-04-17

**Authors:** Faisal Shahzad, Lisa Tang, Tripti Vashisth

**Affiliations:** ^1^ Horticultural Sciences Department, Citrus Research and Education Center, Institute of Food and Agricultural Sciences (IFAS), University of Florida, Lake Alfred, FL, United States; ^2^ United States Department of Agriculture, Agricultural Research Service, Appalachian Fruit Research Station, Kearneysville, WV, United States

**Keywords:** citrus greening, hypoxia, hormones, oxidative stress, ROS production

## Abstract

Devastating citrus disease Huanglongbing (HLB) is without existing cures. Herein, we present results demonstrating the possible mechanisms (hypoxia stress) behind HLB-triggered shoot dieback by comparing the transcriptomes, hormone profiles, and key enzyme activities in buds of severely and mildly symptomatic ‘Hamlin’ sweet orange (*Citrus sinensis*). Within six months (October – May) in field conditions, severe trees had 23% bud dieback, greater than mild trees (11%), with a concomitant reduction in canopy density. In February, differentially expressed genes (DEGs) associated with responses to osmotic stress, low oxygen levels, and cell death were upregulated, with those for photosynthesis and cell cycle downregulated in severe versus mild trees. For severe trees, not only were the key markers for hypoxia, including anaerobic fermentation, reactive oxygen species (ROS) production, and lipid oxidation, transcriptionally upregulated, but also alcohol dehydrogenase activity was significantly greater compared to mild trees, indicating a link between bud dieback and hypoxia. Tricarboxylic acid cycle revival, given the upregulation of *glutamate dehydrogenase* and *alanine aminotransferase* DEGs, suggests that ROS may also be generated during hypoxia-reoxygenation. Greater (hormonal) ratios of abscisic acid to cytokinins and jasmonates and upregulated DEGs encoding NADPH oxidases in severe versus mild trees indicate additional ROS production under limited oxygen availability due to stomata closure. Altogether, our results provided evidence that as HLB progresses, excessive ROS produced in response to hypoxia and during hypoxia-reoxygenation likely intensify the oxidative stress in buds leading to cell death, contributing to marked bud and shoot dieback and decline of the severely symptomatic sweet orange trees.

## 1 Introduction

Huanglongbing (HLB; citrus greening disease) has become a significant threat to citriculture. HLB is presumably caused by a phloem-limited bacterium *Candidatus* Liberibacter asiaticus (*C*Las) and vectored by Asian citrus psyllid (*Diaphorina citri* Kuwayama) respectively (Bové, 2006). To date, there is no cure for HLB. Unfortunately, no known citrus germplasm is resistant to HLB. In Florida, citrus production has declined by 73% in the last fifteen years due to HLB (USDA, 2020). Following *C*Las-infection, dieback of the feeder roots occurs before the prominence of HLB symptoms on the aboveground tree canopy, limiting nutrients and water uptake (Johnson et al., 2014; Shahzad et al., 2020). With HLB progression, leaves show blotchy mottles, nutrient deficit, phloem plugging, and starch accumulation (Bové, 2006). Altogether, leaf drop and twig dieback result in canopy loss, leading to yield losses.

Canopy density is the key indicator to assess HLB severity; tree productivity and fruit drop have been directly correlated with the canopy density of HLB-affected trees (Tang and Vashisth, 2020). It is noteworthy that HLB severity increases over time, and affected trees become non-productive within seven years of infection (Stover et al., 2016). A thinner canopy causes more intense sunlight exposure on vegetative parts and to the ground; thus, an increase in temperature affects the respiration rate of the tree body (Griffin et al., 2002). Since shoot (bud) dieback signifies the first step in the rapid aboveground decline of affected trees, a better understanding of the underlying mechanism will help mitigate or decelerate the progression of HLB symptoms. The basic understanding behind HLB-triggered bud dieback in citrus trees still needs to be discovered. So this study aims to understand the underlying canopy bud dieback mechanism at the physiological, molecular, and hormonal levels in HLB-affected ‘Hamlin’ and ‘Valencia’ sweet orange trees.

## 2 Materials and methods

### 2.1 Plant materials

Fifteen-year-old early-maturing ‘Hamlin’ and seventeen-year-old late-maturing ‘Valencia’ sweet orange trees grafted on ‘Swingle’ citrumelo (*Citrus paradisi* × *Poncirus trifoliata*) rootstock grown in an experimental citrus grove located at the University of Florida Citrus Research and Education Center, Lake Alfred were used in this study. Almost 100% of citrus trees are *C*Las-infected in Florida, so it is implausible to find HLB-free (healthy) citrus trees in the open field for comparison. Therefore, citrus trees (n = 4) exhibiting severe and mild visual symptoms of HLB, i.e., blotchy mottling on leaves and branch dieback (Slinski, 2016), were selected for comparison. Based on the amount of light passing through the canopy to estimate canopy density, photosynthetic photon flux density was higher in severe (625 µmol m^-2^ s^-1^) than mild trees (150 µmol m^-2^ s^-1^) at the start of experiment, indicating ≈ a 4-fold thinner canopy in severe trees.

### 2.2 Physiological attributes

Five terminal buds of 20 non-bearing shoots (100 buds/tree) tagged in advance of the experiment were used to assess bud dieback over time; quiescent (dormant), viable (floral or vegetative), and dead buds were counted monthly from November 2018 through January 2019 and every two weeks from February 2019 through anthesis in March 2019. Given the correlation between canopy density and HLB severity (lower density indicating a greater extent of branch dieback because of HLB), canopy density is an important trait to represent tree health regarding HLB severity. To estimate canopy density, a line quantum meter with the detecting spectrum of photosynthetically active radiation (MQ-306; Apogee Instrument, Logan, UT) was used to quantify light interception by the canopy, expressed as photosynthetic photon flux density (PPFD), in the beginning, and end of the experiment (October 2018 and May 2019, respectively). Measurements were done at solar noon on a clear sky day with the line quantum meter placed underneath the canopy (10 cm above the ground) for both sides of trees; in-row readings (without tree shading) were taken at the same height following each tree measurement. The light interception was calculated by dividing the below-canopy reading by the in-row reading. The % change in PPFD is reported herein, showing the percent increase in light penetrating through the canopy between Oct 2018 and May 2019 measurement (means fewer leaves to intercept light, thinner canopy). Leaf nutrients analysis was performed on 30 leaves with intact petioles from non-bearing branches randomly selected per tree in January 2019, using the protocols described in Shahzad et al. (2020). Fruit yield was determined by hand harvesting ‘Hamlin’ and ‘Valencia’ trees on January 8 and March 10, 2019, respectively, and expressed as total weight (in kilograms) per tree.

Transcriptomic analysis and phytohormones quantification were performed only on ‘Hamlin’ trees exhibiting severe and mild HLB symptoms, and bud samples were collected at two-time points: November 2018, when the buds were dormant, and February 2019, when floral and vegetative buds started active growth. Five terminal buds from ten non-fruiting branches were collected from all four quadrants of the tree. Collected buds from each tree were pooled together and flash-frozen with liquid nitrogen and stored at −80 °C in the freezer until transcriptome, hormonal, and alcohol dehydrogenase enzyme (ADH) analyses.

### 2.3 Transcriptome analysis

Total RNA was extracted from ground bud tissues (100 mg) using the RNeasy Plant Mini Kit (Qiagen, Valencia, CA). RNA quantity and quality were determined using a spectrophotometer and denaturing formaldehyde agarose gels (1.2%) (Rio, 2015), respectively. Total RNA samples were sent to the Interdisciplinary Center for Biotechnology Research, University of Florida (Gainesville) for global transcriptome analyses. The Illumina TruSeq mRNA protocol (Illumina, San Diego, CA) was used for transcripts sequencing. The *C. sinensis* genome v1.1 (Wu et al., 2014) from the Phytozome database was used as the reference. Differential gene expression was analyzed using RSEM v1.2.31 (Li and Dewey, 2011) with a false discovery rate (FDR)-corrected probability value of less than 0.05 (as threshold cutoff). Multiple tools, including MapMan software version 3.5.1.R2 (Usadel et al., 2005), AgriGO in tandem with REVIGO (Tian et al., 2017), and Kyoto Encyclopedia of Genes and Genomes (KEGG) Mapper tool (Kanehisa et al., 2017) were used for the interpretation of the biological significance from RNA seq results as described in Shahzad et al. (2020)

Results obtained from RNA seq analysis were also validated using real-time quantitative PCR (qPCR) following the protocols described by Tang and Vashisth (2020), and gene-specific primers sequences are listed in **Supplemental Table S1**. Eight DEGs related to hormones and cell wall metabolism were analyzed with citrus *actin* and *Thioredoxin-like protein YLS8* as reference genes (Tang and Vashisth, 2020).

### 2.4 Phytohormones quantification

The ground bud samples (also used for RNA seq analysis) were sent to the Proteomics and Metabolomics Facility, Nebraska Center for Biotechnology, the University of Nebraska-Lincoln for the phytohormonal profile of bud. Hormones and their derivatives, including cytokinins (cZ, tZ, cZR, tZR), auxins (IAA, IAA-Ala, IAA-Asp, Methyl-IAA, IAA-Trp), abscisic acid (ABA), jasmonates (OPDA, JA, JA-Ile), salicylic acid (SA), strigolactones, and gibberellins were extracted and analyzed following the protocols described in Hung et al. (2016). Hormonal ratios are presented herein indicate the combined effect between two hormones with antagonistic or mutually similar effects (either stimulating or suppressive).

### 2.5 Alcohol dehydrogenase activity

ADH activity was quantified to assess the rate of alcoholic fermentation, a key marker for hypoxia stress. Buds collected monthly from November 2018 to February 2019 were used to determine ADH activity, following the protocols described by Gonçalves et al. (2010). The results for ADH activity presented herein are in µmol min^-1^ g^-1^ FW.

### 2.6 Statistical analysis

Physiological variables, phytohormone levels, and ADH activity were analyzed to determine the differences between mild and severe trees using t-test in Sigma Plot (version 12; Systat Software, San Jose, CA) with a significance level of α ≤ 0.05.

## 3 Results

### 3.1 Physiological attributes

**Figure 1:**
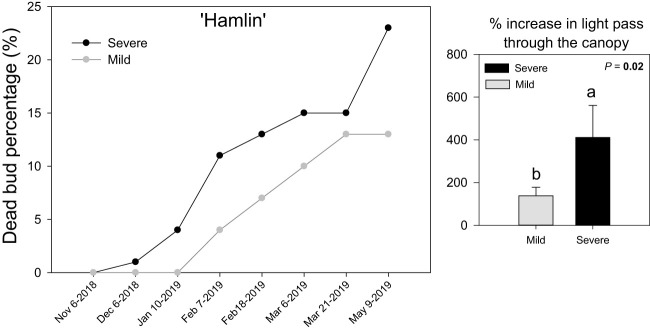
Dead bud percentage (%) on individual survey days and % increase in light penetrating through the canopy (PPFD, µmole/m^2^s) at the end of the experiment in Huanglongbing (HLB)-affected ‘Hamlin’ sweet orange trees with severe and mild symptoms, respectively. Each point is the mean ± standard deviation of four biological replicates. Significant differences were considered when P < 0.05 using Tukey’s honestly significant difference (HSD) test. Different letters indicate significant differences in the canopy density between severe and mild trees.

For both severe and mild ‘Hamlin’ sweet orange trees, shoots with zero dead buds were tagged before the beginning of the experiment (November 2018) (**Figure 1**). ‘Hamlin’ severe trees showed a progression in dead bud percentage starting a month later (December 2018), whereas mild trees did not show such a pattern until January 2019. Overall severe trees underwent significantly more bud dieback than mild trees. This increase in dead bud percentage in ‘Hamlin’ severe trees also coincided with more light penetrating through the canopy to the ground in May 2019, indicating sparse canopies compared to mild trees. Fruit yield was almost 50% lower in ‘Hamlin’ severe trees than in mild trees (**Supplemental Figure S2A**). No differences were found in leaf nutrients concentration in January 2019, floral and vegetative intensity per shoot (on each survey date) between severe and mild ‘Hamlin’ trees (**Supplemental Figure S2B, C**). A similar trend regarding physiological attributes was found in ‘Valencia’ trees exhibiting mild and severe trees (**Supplemental Figure S3A, B, C**).

### 3.2 Transcriptome analysis

In buds of severe and mild ‘Hamlin’ trees collected in November 2018, there were only four differentially expressed genes (DEGs). Among these three DEGs [encoding LOB domain-containing protein 41 (orange1.1g022591m), B12D protein (orange1.1g048115m), and detoxification superfamily protein (orange1.1g033335m)] were upregulated, and one DEG encoding beta-glucosidase 11 (orange1.1g046009m) was downregulated in severe trees compared to mild trees (**Table 1**). These DEGs have been reported to be induced under low oxygen levels in plants (Liu et al., 2005; Lokdarshi, 2015).

**Table 1:** Differentially expressed genes (DEGs) in buds of severely symptomatic Huanglongbing (HLB)-affected ‘Hamlin’ sweet orange trees compared to mildly symptomatic trees in November.

DEGs (*Citrus sinensis*)	*Arabidopsis thaliana* ortholog	Log Fold Change	Description	Functions
orange1.1g022591m	AT3G02550	1.85	LOB domain-containing protein 41	Communication establishment between meristems and initiation of shoots or roots
orange1.1g048115m	AT3G29970	23.9	B12D protein	Germination protein-related similar to HvB12D
orange1.1g033335m	AT3G07600	3.53	detoxification superfamily protein	Metal ion binding
orange1.1g046009m	AT1G02850	-1.79	beta glucosidase 11	Glycosyl hydrolase family protein; induced under biotic and abiotic stresses

In buds collected in February 2019, 2063 genes were differentially expressed between severe and mild trees. Among these, 982 and 1081 DEGs were downregulated and upregulated in the buds of severe trees compared to mild trees. There exists a significant and positive correlation (R = 0.78) between fold changes attained by RNA-seq analysis and relative gene expression levels found by qPCR (data not shown), indicating the validity of RNA-seq data.

#### 3.2.1 Functional analysis of DEGs

**Figure 2:**
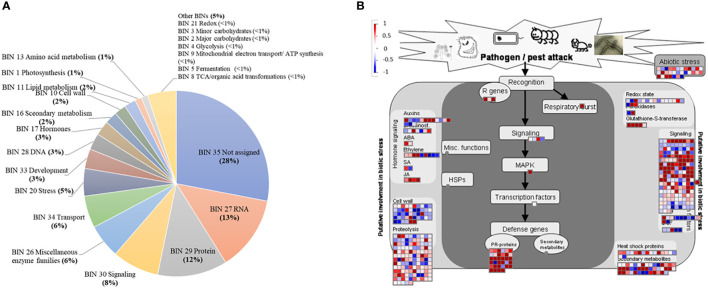
Major functional categories (BINs) using the MapMan enrichment analysis **(A)**; differentially expressed genes in pathogen/pest attack category **(B)**, in buds of severely symptomatic Huanglongbing (HLB)-affected ‘Hamlin’ sweet orange trees compared to mildly symptomatic trees in February. This pathogen/pest attack category includes BINs related to hormones, redox reactions, defense genes, signaling, heat shock proteins, and biotic stress. Red and blue boxes represent upregulated and downregulated genes, respectively.

MapMan enrichment analysis provides an overview of DEGs involved in metabolic and physiological processes in the bud of severe trees compared to mild trees in Feb 2019 (**Figure 2A**). A significant proportion of the DEGs (28%) came under the category with no assigned function (Bin 35), followed by RNA (BIN27; 13%), protein (BIN29; 12%), signaling (BIN30; 8%), miscellaneous enzyme families (BIN26; 6%), transport (BIN34; 6%), stress (BIN20; 5%), development (BIN33; 3%), DNA (BIN28; 3%), hormones (BIN17; 3%), secondary metabolism (BIN16, 2%), cell wall (BIN10, 2%), lipid metabolism (BIN11; 2%), photosynthesis (BIN1; 1%), and amino acid metabolism (BIN13; 1%), with the categories that comprise less than 1% of the DEGs.

Using AgriGO and REViGO tools, gene ontology (GO) enrichment analysis was also performed with the downregulated and upregulated DEGs separately. Significant GO terms for biological processes in the bud of severe trees compared to mild trees (**Table 2**) and cellular processes and molecular functions are listed in **Supplemental Table S2**. In buds of severe versus mild trees for downregulated DEGs, top enriched GO terms were regulation of circadian rhythm (GO:0042752), response to blue light (GO:0009637), photosynthesis (GO:0015979), cell cycle (GO:0007049), cell division (GO:0051301), tissue development (GO:0009888), and auxin transport (GO:0060918) (**Table 2**; **Supplemental Table S3**). For the GO terms associated with molecular functions, chlorophyll binding (GO:0016168), ATP binding (GO:0005524), and carbohydrate phosphate activity (GO:0019203) were the most enriched in downregulated DEGs in severe trees compared to mild trees (**Supplemental Table S2**). For upregulated DEGs in severe versus mild trees, top enriched GO terms included abiotic stress [response to osmotic stress (GO:0006970), response to decreased oxygen levels (GO:0036293)], biotic stress [response to bacterium (GO:0009617), response to chitin (GO:0010200)], defense response, incompatible interaction (GO:0009814), oxidation-reduction process (GO:0055114), aging (GO:0007568), cell death (GO:0008219), hormones [ethylene-activated signaling pathway (GO:0009873) and jasmonic acid biosynthesis process (GO:0009695)] (**Table 2**). Considering molecular functions, dioxygenase activity (GO:0051213) and oxidoreductase activity (GO:0016491) were the most enriched terms in upregulated DEGs of severe trees than mild trees (**Supplemental Table S2**).

**Table 2:** A subset of GO categorization relating to the biological process of downregulated and upregulated differentially expressed genes (DEGs) in buds of severely symptomatic Huanglongbing (HLB)-affected ‘Hamlin’ sweet orange trees compared to mildly symptomatic trees in February.

GO term	Description	DEGs (no.)	ES^z^	*P* value
Down-regulated DEGs				
GO:0042752	regulation of circadian rhythm	8	4.20	1.20E-03
GO:0009639	response to red or far-red light	24	3.12	3.10E-06
GO:0009637	response to blue light	14	4.99	3.30E-06
GO:0009642	response to light intensity	18	3.61	8.90E-06
GO:0015979	photosynthesis	35	3.99	5.30E-11
GO:0048869	cellular developmental process	54	1.56	1.40E-03
GO:0007049	cell cycle	64	3.31	1.90E-15
GO:0051301	cell division	35	2.82	1.80E-07
GO:0006725	cellular aromatic compound metabolic process	228	1.44	1.30E-08
GO:0009653	anatomical structure morphogenesis	51	1.60	1.10E-03
GO:0009888	tissue development	44	2.20	3.00E-06
GO:0006810	transport	113	1.36	6.80E-04
GO:0006820	anion transport	18	2.29	1.60E-03
GO:0010817	regulation of hormone levels	20	2.3	8.70E-04
GO:0060918	auxin transport	12	3.47	0.00038
GO:0006793	phosphorus metabolic process	99	1.63	2.60E-06
Upregulated DEGs				
GO:0006950	response to stress	226	1.69	8.10E-15
GO:0006970	response to osmotic stress	42	1.73	7.50E-04
GO:0036293	response to decreased oxygen levels	10	3.73	6.80E-04
GO:1901700	response to oxygen-containing compound	97	1.63	3.60E-06
GO:0009617	response to bacterium	51	3.03	3.30E-11
GO:0010200	response to chitin	15	2.93	0.0004
GO:0009611	response to wounding	20	2.41	5.10E-04
GO:0006952	defense response	135	2.26	1.60E-17
GO:0009814	defense response, incompatible interaction	18	2.77	0.00021
GO:0045087	innate immune response	30	2.43	2.00E-05
GO:0055114	oxidation-reduction process	89	1.49	0.00021
GO:0008219	cell death	27	3.65	5.00E-08
GO:0009725	response to hormone	98	1.57	1.30E-05
GO:0009873	ethylene-activated signaling pathway	20	2.83	7.30E-05
GO:0009695	jasmonic acid biosynthetic process	6	5.81	1.10E-03
GO:0048856	anatomical structure development	154	1.28	1.00E-03
GO:0051093	negative regulation of the developmental process	14	2.93	6.20E-04
GO:0007568	aging	20	3.01	3.40E-05
GO:0006793	phosphorus metabolic process	128	1.92	8.50E-12
GO:1901698	response to nitrogen compound	22	2.18	9.50E-04

^Z^ Enrichment score (ES) for individual GO terms was determined by: (DEG number in the GO term/total DEG number)/(number of DEGs in the genes in the genome for the GO terms/total genes number in the genome).

#### 3.2.2 Response to decreased oxygen levels (hypoxia stress)

In severe trees, 42 DEGs related to osmotic stress (GO:0006970) and 10 DEGs related to decreased oxygen levels (GO:0036293) were upregulated compared to mild trees (**Table 2**; **Supplemental Table S4**). DEGs known to be induced under hypoxia stress and in the hypoxia-reoxygenation phase are listed in **Table 3**. Anaerobic fermentation (ethanol) is the critical metabolic adaptation to confirm energy production in hypoxic conditions (Liu et al., 2005; Bui et al., 2015; León et al., 2021); DEG encoding pyruvate decarboxylase (orange1.1g007800m) and those encoding alcohol dehydrogenase (orange1.1g035170m, orange1.1g047713m) were upregulated in severe trees than mild trees. For the starch/sucrose utilization process, *alpha-amylase-like 3* (orange1.1g002585m), *hexokinase 2* (orange1.1g010895m), and *phosphofructokinase 2* (orange1.1g042534m) genes were upregulated in severe trees than mild trees. Since hypoxia hampers the mitochondrial activity and plays a role in an anoxic burst of cytosolic calcium (Ca) (Subbaiah and Sachs, 2003; León et al., 2021), a DEG encoding autoinhibited Ca^2+^-ATPase 1 (orange1.1g016687m) was upregulated in severe trees than mild trees. Regarding low oxygen sensing mechanism, DEGs encoding group VII ethylene response factor family (ERFVIIs) (orange1.1g017035m, orange1.1g019887m) and two *shikimate kinase* genes (orange1.1g023118m, orange1.1g025114m) were upregulated in severe trees than mild trees. Ethylene-regulated DEGs encoding enzymes alanine aminotransferase (orange1.1g009517m) and glutamate dehydrogenase (orange1.1g012750m) were upregulated in severe trees compared to mild trees, indicating reoxygenation following hypoxia. Related to hormones, DEGs encoding cytokinin oxidase (orange1.1g009793m, orange1.1g008291m) and gibberellin 2-oxidase 1 (orange1.1g020152m) were upregulated in severe trees than mild trees. DEG encoding NADPH oxidases (orange1.1g002711m; responsible for ROS generation) were upregulated in severe trees compared to mild trees. During hypoxia, excessive production of ROS from apoplastic NADPH oxidases and then following reoxygenation cause oxidative stress *via* the electron transport chain from mitochondria and chloroplasts, resulting in autophagy (Chen et al., 2017; Qi et al., 2020; León et al., 2021; Foyer and Hanke, 2022). All six autophagy indicator genes were upregulated in severe trees than in mild trees.

**Table 3:** Differentially expressed genes (DEGs) relating to biological processes induced by cellular hypoxia levels and during the hypoxia-reoxygenation phase in buds of severely symptomatic Huanglongbing (HLB)-affected ‘Hamlin’ sweet orange trees compared to mildly symptomatic trees in February.

Biological processes	DEGs (*Citrus sinensis*)	*Arabidopsis thaliana* ortholog	Log Fold Change	Description	Functions
Response to decreased oxygen level (hypoxia)					
***ROS production***					NADPH oxidases generate ROS in plant apoplasts and increase H_2_O_2_ production upon hypoxia.
Respiratory burst	orange1.1g002711m	AT1G09090	2.77	respiratory burst oxidase homolog B	
Alternate oxidase	orange1.1g007736m	AT1G12110	-1.83	nitrate transporter 1.1	AOX-based alternative respiration in the mitochondrial electron transport chain ensures metabolic homeostasis by directly reducing oxygen to water in transient hypoxic conditions.
	orange1.1g014301m	AT5G14570	-1.38	High-affinity nitrate transporter 2.7	
Carbon metabolism					
Anaerobic fermentation	orange1.1g007800m	AT5G54960	1.92	pyruvate decarboxylase 2	Activation of fermentation pathways (through pyruvate decarboxylase and alcohol dehydrogenase enzymes) helps sustain glycolysis without mitochondrial respiration and tissue survival.
	orange1.1g035170m	AT1G77120	3.61	alcohol dehydrogenase 1	
	orange1.1g047713m	AT1G77120	2.16	alcohol dehydrogenase 1	
Carbohydrate metabolism	orange1.1g002585m	AT1G69830	1.49	alpha-amylase-like 3	The utilization of starch or sucrose is essential for hypoxia tolerance. One key step of glycolysis, phosphofructokinase (PFK), is induced under hypoxia that uses PPi rather than ATP to save energy.
	orange1.1g010895m	AT2G19860	1.45	hexokinase 2	
	orange1.1g038564m	AT5G58730	1.60	pfkB-like carbohydrate kinase family protein	
	orange1.1g042534m	AT5G47810	1.38	phosphofructokinase 2	
Hormonal regulation					
Ethylene group VIIs	orange1.1g017035m	AT1G68840	3.18	related to ABI3/VP1 2	Sensitivity to low oxygen levels relies on the stability of the group VII ethylene response factor family (ERFVIIs), which act as an oxygen sensor under low oxygen conditions.
	orange1.1g019887m	AT3G16770	2.69	ethylene-responsive element binding protein	
Gibberellins	orange1.1g020152m	AT1G78440	2.22	*Arabidopsis thaliana* gibberellin 2-oxidase 1	Gibberellic acid metabolism is important for hypoxia followed by the hypoxia-reoxygenation phase.
Cytokinins	orange1.1g009793m	AT5G56970	2.08	cytokinin oxidase 3	Cytokinins regulate growth and senescence and contribute to post-stress recovery upon hypoxia.
	orange1.1g008291m	AT3G63440	1.29	cytokinin oxidase/dehydrogenase 6	
Auxins	orange1.1g032663m	AT1G72430	2.96	SAUR-like auxin-responsive protein family	Low oxygen conditions trigger auxin accumulation, while the following reaeration reduces its concentration
	orange1.1g036854m	AT4G22620	1.95	SAUR-like auxin-responsive protein family	
	orange1.1g034194m	AT5G53590	2.22	SAUR-like auxin-responsive protein family	
***Calcium***					
	orange1.1g016687m	AT1G27770	2.35	autoinhibited Ca^2+^-ATPase 1	Calcium triggers the downstream signal pathways under hypoxia.
Transcription factors (TFs)					
WRKY	orange1.1g020713m	AT2G47260	2.69	WRKY DNA-binding protein 23	Involvement of WRKY helps in post-stress recovery.
	orange1.1g032690m	AT2G47260	1.37	WRKY DNA-binding protein 23	
VQ-motif	orange1.1g033523m	AT1G78410	-2.62	VQ motif-containing protein	VQ proteins regulate growth and development processes under environmental stress.
	orange1.1g029381m	AT2G22880	-2.22	VQ motif-containing protein	
SK1	orange1.1g023118m	AT2G21940	1.56	shikimate kinase 1	*ERFVII*s group involved in the adaptative response to hypoxia consists of N terminal met-Cys motif (*SKI*).
	orange1.1g025114m	AT4G39540	1.31	shikimate kinase 2	
Lateral organ boundary domain protein (LBD)	orange1.1g038007m	AT3G02550	3.56	LOB domain-containing protein 41	Hypoxia hampered the aquaporins activity by lowering cytosolic pH and induction of LBD-related DEGs is important for communication between meristem regions.
	orange1.1g022591m	AT3G02550	3.14	LOB domain-containing protein 41	
Glutamate dehydrogenase	orange1.1g012750m	AT5G07440	1.60	glutamate dehydrogenase 2	During the hypoxia-reoxygenation phase, alanine aminotransferase/glutamate dehydrogenase cycle enzymes resume the tricarboxylic acid cycle.
Alanine aminotransferase	orange1.1g009517m	AT1G72330	1.14	alanine aminotransferase 2	
Autophagy					
	orange1.1g018248m	AT3G62770	1.52	transducin/WD40 repeat-like superfamily protein	Hypoxia induces and accelerates autophagosome formations.
	orange1.1g041604m	AT3G51790	1.57	transmembrane protein G1P-related 1	
	orange1.1g019349m	AT4G30510	1.19	homolog of yeast autophagy 18 (ATG18) B	
	orange1.1g002446m	AT5G54730	1.34	homolog of yeast autophagy 18 (ATG18) F	
	orange1.1g022503m	AT3G51840	2.73	acyl-CoA oxidase 4	
	orange1.1g033386m	AT1G62040	1.33	ubiquitin-like superfamily protein	

#### 3.2.3 DEGs related to stress

GO terms related to stress (GO:0006950) and oxidation-reduction process (GO:0055114) were upregulated in severe trees than in mild trees (**Table 2**). MapMan category for tree-pathogen/pest interactions was highly enriched, which consists of redox, hormones metabolism, cell wall, pathogenesis-related (PR) proteins, secondary metabolism, transcription factors, and abiotic stresses (**Figure 2B**; **Supplemental Table S5** and **S6**). In the case of the redox category, two and 11 DEGs related to *glutathione peroxidase* and *glutathione S transferase*, respectively, were upregulated in severe trees than in mild trees (**Supplementary Table S5**). Fatty acid beta-oxidation-related DEGs (11) were upregulated in severe trees compared to mild trees (**Supplemental Table S6**), indicating oxidative stress. Consistently, the GO term for cell death (GO:0008219) was upregulated in severe trees compared to mild trees (**Supplemental Table S4**). In addition, DEGs related to stress-induced hormones ABA and JA biosynthesis processes were upregulated in severe trees compared to mild trees (**Supplemental Table S6**).

### 3.3 Phytohormones quantification

**Figure 3:**
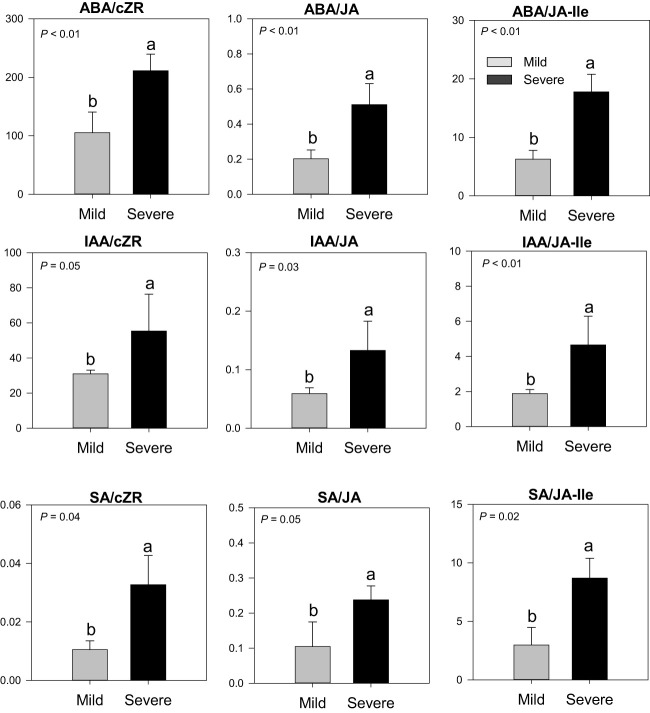
Phytohormones ratios (ABA, IAA, JA, JA/Ile, SA, and cZR) in buds of Huanglongbing (HLB)-affected ‘Hamlin’ trees. Data are presented as means ± standard deviation (n=4). Different letters marked on bars indicate significant differences among severe and mild trees. Grey and black colors represent mild and severe trees, respectively. ABA, abscisic acid; JA, jasmonic acid; JA-Ile, jasmonic acid iso leucine; IAA, auxins; SA, salicylic acid; cZR, cytokinins.

In buds collected in February 2019, severe trees had higher ratios of ABA/cZR (2-fold), ABA/JA (2.5-fold), ABA/JA-Ile (3-fold), IAA/cZR (1.8-fold), IAA/JA (2.3-fold), and IAA/JA-Ile (2.5-fold) in comparison to mild trees (**Figure 3**). The SA/JA (2.3-fold), SA/JA-Ile (2.9-fold), and SA/cZR (3-fold) ratios were also higher in severe trees than in mild trees. No such differences were found in the phytohormones ratios in the bud of severe and mild trees in November 2018 (data not shown). Furthermore, gibberellins and strigolactones were not detected in bud samples at any time.

### 3.4 Alcohol dehydrogenase activity

**Figure 4:**
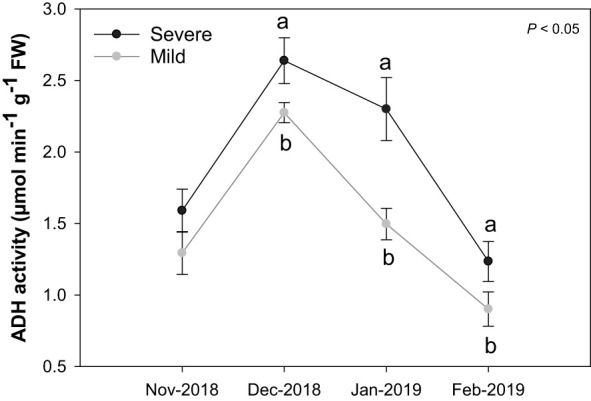
Alcohol dehydrogenase enzyme activity (µmol min^-1^ g^-1^ FW) in buds of Huanglongbing (HLB)-affected ‘Hamlin’ trees. Data are presented as means ± standard deviation (n=4). Different letters indicate significant differences among severe and mild trees. Grey and black colors represent mild and severe trees, respectively.

In December 2018, both mild and severe trees had the maximum ADH activity (**Figure 4**). In January 2019, ADH activity decreased rapidly in mild trees (when active growth started). In contrast, severe trees retained high ADH activity similar to the mild trees’ level in December 2018. A similar pattern for ADH activity between mild and severe trees was found in February 2019. Overall, severe trees had higher ADH activities than mild trees at all-time points.

## 4 Discussion

This study presents multiple lines of evidence regarding physiochemical and transcriptional regulation for bud dieback in HLB-affected sweet orange trees for the first time. Following *C*Las-infection, branches of citrus trees start to die back, leading to low productivity in a few years (Stover et al., 2016). Our results showed that both ‘Hamlin’ and ‘Valencia’ displaying severe HLB symptoms undergo higher bud dieback than mild trees. Additionally, the upregulation of DEGs related to osmotic stress in severe trees than in mild trees indicated limited nutrient and water uptake, which is a hallmark of HLB-affected trees as a result of the reduction in root-to-shoot ratio, root longevity, leaf area, and stomatal conductance compared with healthy trees (Hamido et al., 2019; Shahzad et al., 2020; Silva et al., 2021). Reduced photosynthesis and increased respiration have been reported in HLB-affected trees compared to healthy trees (Silva et al., 2021). Moreover, HLB-affected leaves have lower predawn and midday stem water potential (by 13%) due to less stomatal conductance than healthy trees, directly influencing the stomatal conductivity (Hamido et al., 2019; Silva et al., 2021), thus, gas exchange and water uptake efficiency.

Stomatal oscillations determine transpiration and are modulated by a network of phytohormones (Song et al., 2006; Melotto et al., 2017). Our results showed higher ABA/cZR, ABA/JA, IAA/cZR, and IAA/JA ratios in buds of severe trees than in mild trees in February. Water deficit results in ABA accumulation which initiates signals for stomatal closure resulting in decreased water conductivity and transpiration (Stoll et al., 2000; Tanaka et al., 2006). Water deficit also inhibits cytokinin synthesis in roots and its translocation in the shoot system, whereas higher cytokinins can suppress stomatal closure induced by ABA and help to regulate transpiration (Stoll et al., 2000; Song et al., 2006). Auxin typically stimulates stomatal opening and suppresses ABA-induced stomatal closure (Daszkowska-Golec and Szarejko, 2013); however, excessive auxin accumulation can result in stomatal closure (Qi et al., 2020), as high auxin accumulation occurs under drought/hypoxia stress (León et al., 2021). A similar trend of high IAA and ABA ratios to other hormones was found in severe trees compared to mild trees. HLB-affected trees have been reported to have higher SA accumulation (Nehela et al., 2018); in this study, severe trees also had higher SA/cZR, SA/JA, and SA/JA-Ile ratios than those mild trees. SA stimulates stomatal closure, while cZR and JA induce stomatal opening (Song et al., 2006). Hormone-dependent stomatal oscillation plays a role in pathogen-associated molecular patterns (PAMPs) in *Arabidopsis*. According to a PAMP model by Melotto et al. (2017), in the first phase of PAMPs, stomata close regarding changes in ABA levels within an hour to limit pathogen entry. In the second phase, stomata open after 3 to 4 hours in a JA-dependent manner. Due to the recognition of bacterial pathogens, the third phase starts when stomata close again in association with SA. Similarly, alterations in hormone balances have been documented in ‘Valencia’ sweet orange upon *C*Las infection (Martinelli et al., 2013; Nehela et al., 2018). Taken together, it is likely that the changes in hormone ratios (high SA, ABA, and auxin and low cZR and JA) observed in buds of severe trees contributed to a greater extent of stomatal closure compared with mild trees. All the DEGs involved in the JA biosynthesis process were upregulated in severe trees compared to mild trees (**Supplementary Table 5**). However, the ratios of JA and its derivatives (JA-Ile) were lower in severe trees. A possible explanation of such discrepancy is that the JA biosynthesis process occurs in different cell parts involving chloroplast, peroxisome, and cytoplasm (**Supplemental Figure S4**) (Miersch et al., 2008). In later synthesis stages, JA in the cytoplasm is converted into the derivative compounds [Me-JA, JA-Ile, and 12-hydroxyjasmonic acid (12-OH-JA)]. In our study, 3 DEGs encoding the enzyme for 12-OH-JA were upregulated in severe trees than mild trees, which is an inactive form of JA and responsible for negative feedback mechanism through activation of jasmonate ZIM domain (JAZ) repressor genes by JA (Miersch et al., 2008). Finally, a possible switch-off mechanism behind upregulated genes for JA signaling coincides with the hydroxylation of JA.

Reduced photosynthesis in HLB-affected trees (Silva et al., 2021) and hormonal-induced stomatal closure are indicators of the reduced availability of oxygen at the cellular level. Trees uptake mineral nutrients and oxygen from the soil *via* roots (Cannon, 1932). Oxygen availability provides the most energy in plants and animals, which is crucial for the sustainability of most life forms (Schmidt-Rohr, 2020). Hypoxia act as a double-edged sword. Developmental hypoxia is critical during dormancy to preserve genome integrity by maintaining quiescence within meristems (Considine et al., 2017). On the other hand, hypoxia stress can occur during growth and development phases; metabolically active cells such as developing meristems, including shoots or root apical zones, are partially liable to oxygen deficiency due to higher oxygen demand and less intracellular spaces to conduct oxygen *via* gas diffusion (Drew, 1977). Hypoxia cores can develop when internal oxygen concentrations fall short of the ambient oxygen levels, even though oxygen is available externally (Subbaiah and Sachs, 2003). It is also noteworthy that within all bacteria, including gram-negative *C*Las, energy acquisition by the electron transport chain and ATP synthase for multiplication relies on oxygen (Boyer, 1997). So, it is possible that severely HLB-affected trees would have more oxygen demand than available supply resulting in the development of more hypoxia cores within the plant, thus, intensifying HLB severity.

Transcriptomic results and ADH activity also indicate that severe trees were likely undergoing hypoxia stress. Under hypoxia, the mitochondrial respiratory activity is hampered, and the resulting low ATP level triggers the upregulation of genes responsible for energy production, including fermentation, regulation of metabolic processes, and transcription factors, to cope with the accumulation of toxic products (lactate, or acetaldehyde/ethanol) and ROS (Liu et al., 2005; Bui et al., 2015; León et al., 2021). ROS production and fermentation are key indicators of hypoxia stress (León et al., 2021). NADPH oxidases (also known as respiratory burst oxidase homologs; RBOHs) generated ROS in plant apoplasts as documented in different crops (Hong et al., 2020), including HLB-affected sweet orange (Ma et al., 2022) and increased H_2_O_2_ production in response to hypoxia (Mori et al., 2001; Pasternak et al., 2005; Hong et al., 2020). Ethanol fermentation ensures energy production under low oxygen conditions. In the current study, DEGs encoding alcohol dehydrogenase and pyruvate decarboxylase were upregulated in severe trees compared to mild trees. Concomitantly, higher alcohol dehydrogenase (ADH) enzyme activity was seen in severe trees compared to mild trees. ADH activity increases to meet the energy demand through ATP synthesis under hypoxic as documented in different crops (Sousa and Sodek, 2002; Gonçalves et al., 2010). In citrus, an *ADH* gene was upregulated in HLB-affected trees compared to healthy trees (Zhu et al., 2020), suggesting *C*Las-infection responses involving ADH. In *Arabidopsis*, hypoxia causes an anoxia burst of cytosolic Ca, which acts as a messenger to regulate ion homeostasis activating the downstream signaling pathway following low oxygen conditions (Subbaiah and Sachs, 2003; León et al., 2021). Hypoxia also hampers the aquaporin’s activity by lowering the cytosolic pH and downregulating DEGs encoding lateral organ boundary domain protein (LBD) that are important for communication between the cells within meristem regions (Liu et al., 2005). Sensitivity to low oxygen levels relies on the stability of the *group VII ethylene response factor family* (*ERFVII*), which are oxygen sensors in plants by the N-end rule pathway for protein degradation (Sasidharan and Mustroph, 2011; Bui et al., 2015), and their expression decreases with reoxygenation. The involvement of *ERFVIIs* with N terminal met-Cys motif (*SK1*) induces hypoxia tolerance (León et al., 2021), and these DEGs (orange1.1g023118m, orange1.1g025114m) were upregulated in severe trees than mild trees. Furthermore, alternative oxidase (AOX) is essential to protect lipids from oxidative stress (e.g., nitric oxide and superoxide) under the hypoxia-reoxygenation phase (Jayawardhane et al., 2020), and our results demonstrated the downregulation of AOX in severe in comparison to mild trees, suggesting severe trees underwent a greater level of oxidative stress.

ROS plays a vital role in stress response (biotic and abiotic) along with the developmental process by establishing communication among cells and transducing signals over long distances (Foyer and Hanke, 2022). While hypoxia-reoxygenation mechanisms about ROS production and regulation of transcription factors have been well studied in animals (Mallet et al., 2018), little is known in plants. Plants produce ROS through non-enzymatic (the electron transport chain in chloroplasts and mitochondria) and enzymatic pathways (photorespiration in peroxisomes and plasma membrane-localized NADPH oxidases) (Foyer and Hanke, 2022). Our results demonstrated that the upregulation of DEGs encoding alanine aminotransferase and glutamate dehydrogenase enzymes played a critical role in the tricarboxylic acid cycle revival upon reoxygenation following hypoxia (Tsai et al., 2016), resulting in additional ROS formation (Yeung et al., 2019). Furthermore, in plants, the retrieval process from hypoxia also involves a darkness-to-light transition along with reoxygenation which causes a light-induced reduction in photosynthesis capacity followed by photoinhibition, altogether resulting in excessive ROS production (Yeung et al., 2019). Another source of ROS and its accumulation is the plasma membrane-localized NADPH oxidases (RBOHs) which can be activated with higher ABA, auxin, and SA accumulation (Mori et al., 2001; Pasternak et al., 2005). Our results show that severe trees had higher ratios of ABA, auxin, and SA to other hormones and upregulated DEG encoding RBOH compared to mild trees. ROS generated through simultaneous reoxygenation, darkness-to-light transition, and oxidative stresses in the hypoxia-reaeration phase can cause chlorosis due to accelerated chlorophyll degradation (Yeung et al., 2019), a typical HLB symptom that was observed in severe trees in this study. Under stress conditions, excessive ROS accumulation can be detrimental to cells. A recent report also suggests that HLB results in excessive ROS production causing cellular damage in citrus (Ma et al., 2022). Taken together, hypoxia and hypoxia-reoxygenation phases produce and accumulate excessive ROS resulting in oxidative stress and induced cell death in the severe trees.

## 5 Conclusions

Our results provided evidence suggesting that buds of severely affected HLB trees underwent hypoxia, resulting in ≈ 50% greater dieback over time than mild trees. Bud from severe trees had higher ABA/cZR and ABA/JA ratios than mild trees, which likely induced stomatal closure and accentuated osmotic stress. It is possible that the reduction in feeder root biomass, photosynthesis, and transpiration, which corresponded to the severe HLB symptoms at the canopy level, in severe trees, limited oxygen availability at the cellular level than mild trees. Following *C*Las-infection and HLB progression, a high energy demand (for defense and growth) is being compensated by attenuating respiration, a continuous process all day and night. Thus, to efficiently generate and preserve energy, trees switch to anaerobic respiration under low oxygen conditions, but in the long run, it is a waste of available carbohydrate sources, along with excessive ROS production and toxins accumulation (acetaldehyde/ethanol or lactate) as indicated by more dead buds in severe trees than mild trees. Further, severe trees were undergoing hypoxia stress as indicated by a variety of adaptive responses that include shifting to alternate pathways for ATP production and efficient utilization, phytohormones regulation, and antioxidants mechanism to cope with ostensibly higher ROS accumulation. In the hypoxia recovery phase, reoxygenation involves excessive ROS production leading to oxidative stress, resulting in cell death that may be responsible for rapid bud dieback in severe HLB-affected trees compared to mild trees. It should be noted that before the appearance of aboveground HLB symptoms, dieback occurs first at the feeder roots, which are prone to develop hypoxia cores due to highly active metabolism but little internal air space for oxygen diffusion. However, whether there is a direct connection between hypoxia and *C*Las-caused initial root dieback remains unclear. Therefore, further research is needed to explore this relationship as well as the bases for the hypoxia localized in specific tree organs (feeder roots, developing leaves and fruit, etc.) with HLB progression.

## Data availability statement

The datasets presented in this study can be found in onlinerepositories. The names of the repository/repositories and accessionnumber(s) can be found below: NCBI, GSE228890.

## Author contributions

FS executed the experiment, analyzed the data, and wrote the manuscript. LT executed experiments in the field, collected samples, assisted in writing manuscript. TV supervised the study, developed a research goal and experimental plan, provided intellectual support. All authors contributed to the article and approved the submitted version.
