# Supplementary material for: Unraveling the mystery of canopy dieback caused by citrus disease Huanglongbing and its link to hypoxia stress
Source: Front Plant Sci. 2023 Apr 17;14:1119530. doi: 10.3389/fpls.2023.1119530 (PMC10149946; doi:10.3389/fpls.2023.1119530)
Supplement: Supplementary file 1 [file DataSheet_1.docx]

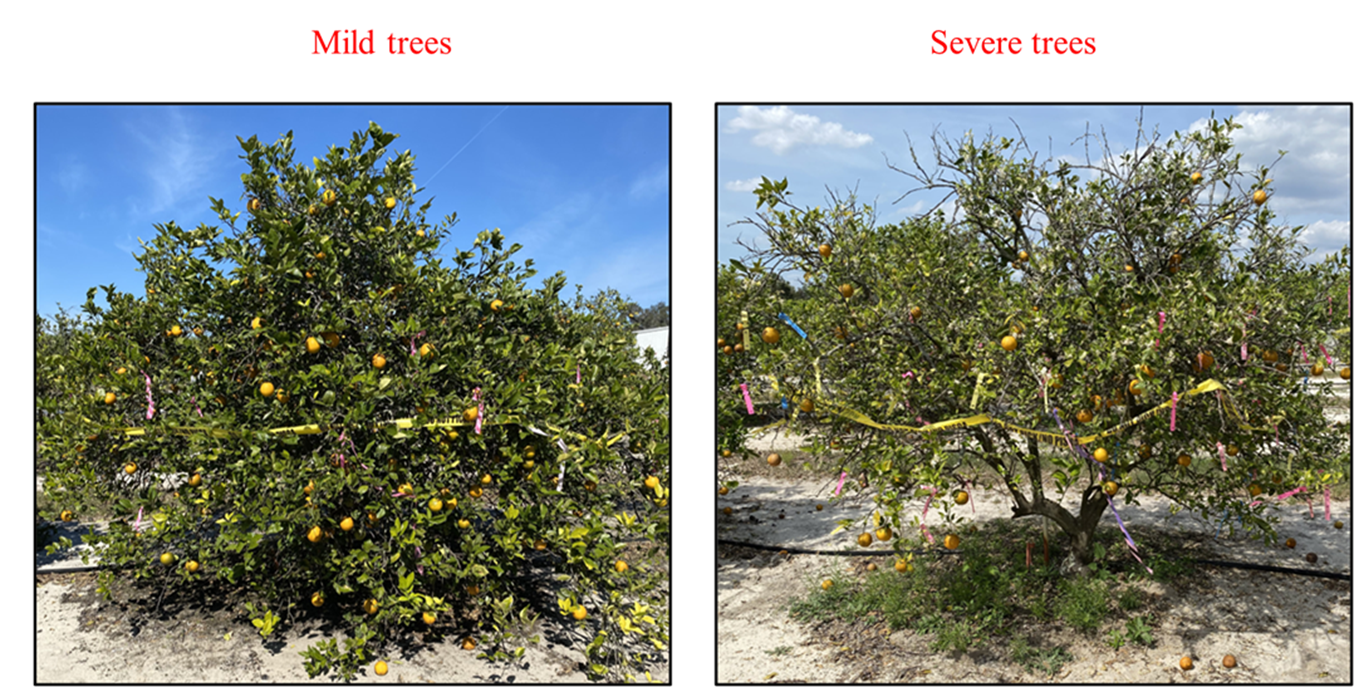


**Supplementary Figure S1.** Tree appearance of ‘Hamlin’ trees as affected by Huanglongbing (HLB) disease exhibiting different severity levels (mild and severe symptoms).


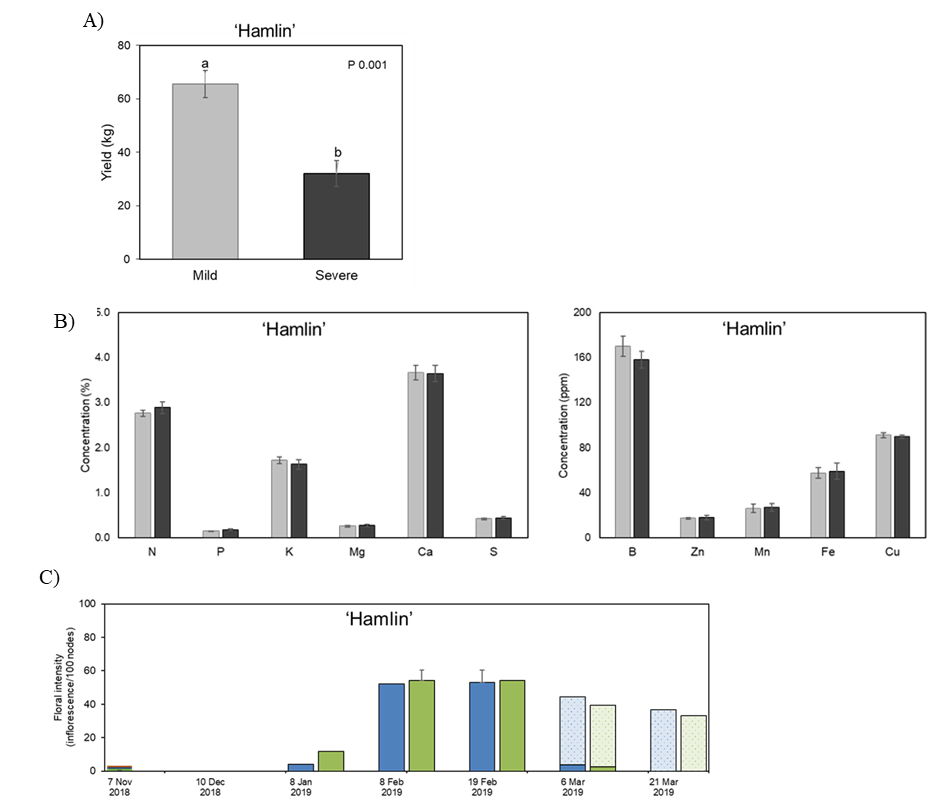


**Supplementary Figure S2.** Fruit yield (A); leaf mineral nutrients concentrations (B); floral intensity (C) in Huanglongbing (HLB)-affected trees ‘Hamlin’ as affected by different HLB severity levels (mild and severe symptoms). Data are presented as the mean±SD of four biological replicates. Grey and black colors represent mild and severe trees, respectively.


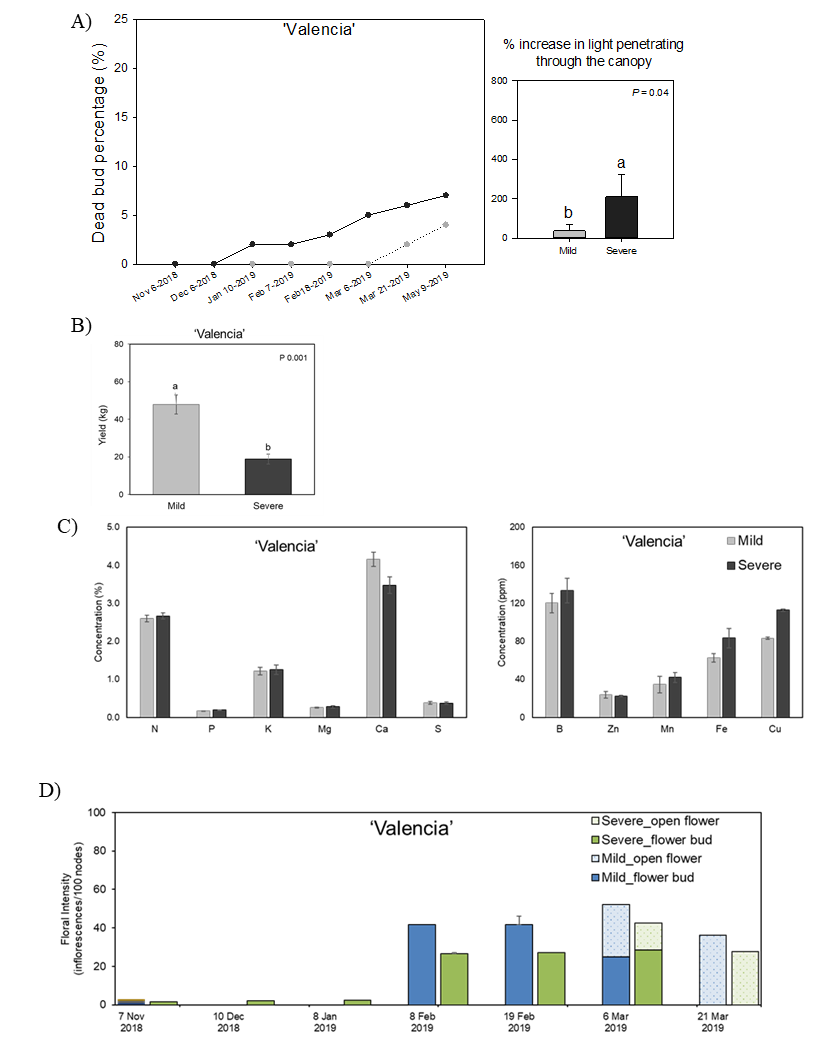


**Supplementary Figure S3.** Dead bud percentage (%) on individual survey days and % increase in light penetrating through the canopy (PPFD, umole/m^2^s) at the end of experiment (A); fruit yield (B); leaf mineral nutrients concentrations (C); floral intensity (D) in Huanglongbing (HLB)-affected trees ‘Valencia’ as affected by different HLB severity levels (mild and severe symptoms). Data are presented as the mean±SD of four biological replicates. Grey and black colors represent mild and severe trees, respectively.

**
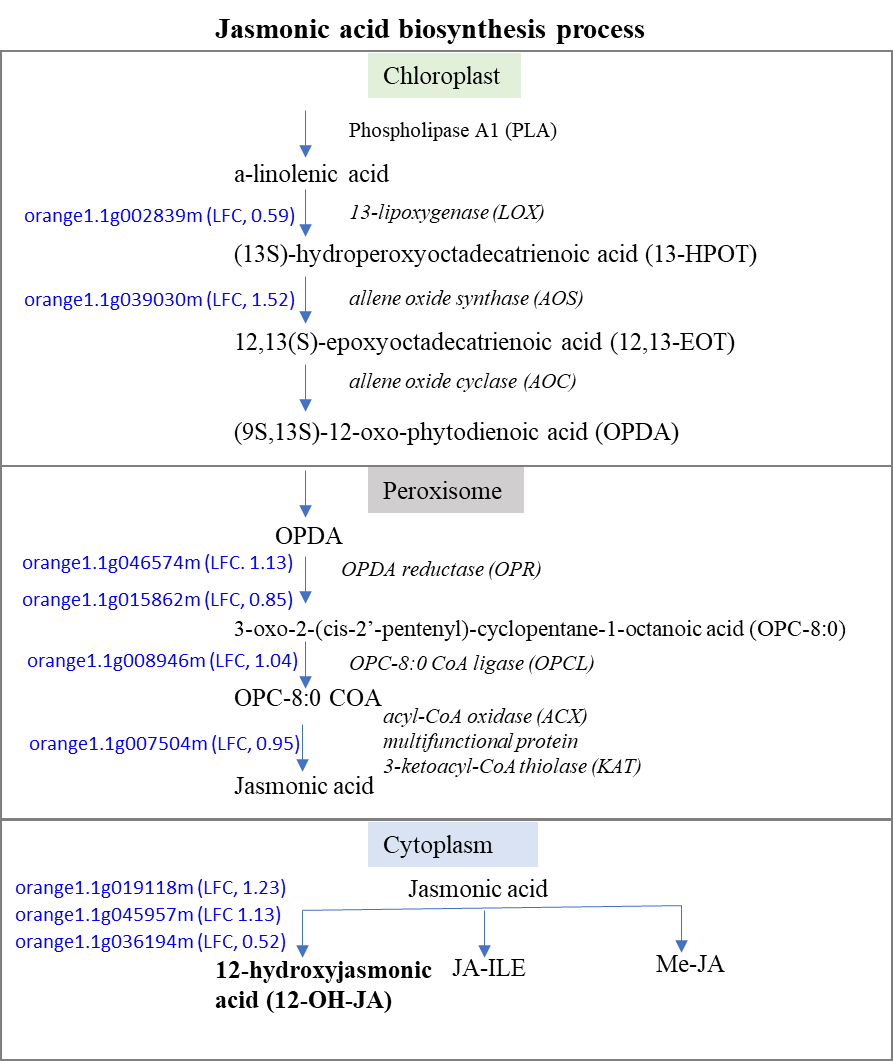
**

**Supplementary Figure S4.** Schematic diagram of jasmonic acid biosynthesis and metabolism in response to biotic stress. Differentially expressed genes (DEGs) presented herein are upregulated in buds of severely symptomatic Huanglongbing (HLB)-affected ‘Hamlin’ sweet orange trees compared to mildly symptomatic trees. JA = jasmonic acid; JA-Ile = jasmonoyl isoleucine; Me-JA = methyl Jasmonate; LFC = log_2_ fold change.

| **Supplementary Table S1**. Gene-specific primer sequences for relative gene expression analysis in buds of severely symptomatic Huanglongbing (HLB)-affected ‘Hamlin’ sweet orange trees compared to mildly symptomatic trees, with quantitative real-time polymerase chain reaction (qPCR) for RNA-sequencing data validation. | | | |
| --- | --- | --- | --- |
| **Gene symbol** | **Description** | **GenBank accession no.** | **Forward and reverse primer sequences (5’ to 3’)** |
| *IAA9* | Indole-2 acetic acid inducible 9 | XM_006466306.3 | ATGGTGACTGGATGCTCGTT |
|  |  |  | TTTTTCCATTGCCCTGGGAG |
| *ARF16* | auxin response factor 16 | XM_006472629.2 | GGAGTTTCGCCACATTTAT |
|  |  |  | TCCATTGTTTCCGTACCT |
| *PP2-B15* | phloem protein 2-b15 | XM_015528590.1 | ATACGCTGCCTACCTCATCGTTAA |
|  |  |  | CGCGATGCAAAAAGTACAGATGCC |
| *ACS1* | ACC synthase for ethylene biosynthesis | NM_001288944.2 | CAAGCTTCTCTAGCCATAAC |
|  |  |  | GACAAGATTCCCATTGTCTC |
| *ERF1* | Ethylene-responsive transcription factor | XM_006488353.3 | GCTGTAGGGTCACAATTATC |
|  |  |  | CACCAAATAGTAGCAGTAGC |
| *ACO1* | ACC oxidase 1 for ethylene biosynthesis | XM_006475390.2 | GGAGCTATTGGACTTGTTATG |
|  |  |  | GTGGGTAGTTGCTAACTTTG |
| *ARF* | Auxin-response factor | NM_001288860.1 | TTCGTGTTGGTGTAAGGCGA |
|  |  |  | TGATGGGCTTGTTCTTGGCT |
| *PG20* | Polygalacturonase 20 | XM_006483616.2 | TGTTCGAGCTACCGATATTA |
|  |  |  | CCTGAAAGTCTTGCTCTATTC |
| *ACT7z* | Actin 7 | XM_006464503.2 | GTTGGTTGACATGGAGAAG |
|  |  |  | GACGGTTGAGTACAGAAATAAG |
| *DIM1* | Thioredoxin-like protein YLS8 | XM_006484463.2 | CGAAACCTGTATGCAGATGG |
|  |  |  | ACGGTTGAGGGATCGTAAAG |
| ^z^*ACT* and *DIM1* were used as internal controls for relative gene expression analysis with qPCR. | | | |

| **Supplementary Table S2**. A subset of GO categorization relating to cellular processes and molecular functions of downregulated and upregulated differentially expressed genes (DEGs) in buds of severely symptomatic Huanglongbing (HLB)-affected ‘Hamlin’ sweet orange trees compared to mildly symptomatic trees in February. | | | | | | |
| --- | --- | --- | --- | --- | --- | --- |
| **GO term** | **Ontology** | **Description** | | **DEGs (no.)** | **ES^z^** | ***P* value** |
| **Down-regulated DEGs** |  |  | |  |  |  |
| Cellular process |  |  | |  |  |  |
| GO:0031225 | C | anchored component of membrane | | 21 | 2.17 | 0.0013 |
| GO:0016021 | C | integral component of membrane | | 269 | 1.46 | 6.40E-11 |
| GO:0031226 | C | intrinsic component of plasma membrane | | 21 | 2.08 | 0.0021 |
| GO:0044436 | C | thylakoid part | | 43 | 2.68 | 2.80E-08 |
| GO:0044444 | C | cytoplasmic part | | 453 | 1.19 | 1.10E-06 |
| GO:0043231 | C | intracellular membrane-bounded organelle | | 700 | 1.11 | 2.10E-06 |
| GO:0044446 | C | intracellular organelle part | | 271 | 1.60 | 1.50E-15 |
| GO:0043232 | C | intracellular non-membrane-bounded organelle | | 79 | 1.48 | 0.00055 |
| GO:1990234 | C | transferase complex | | 29 | 1.88 | 0.0015 |
| Molecular process |  |  | |  |  |  |
| GO:0003824 | F | catalytic activity | | 454 | 1.44 | 9.10E-20 |
| GO:0004871 | F | signal transducer activity | | 27 | 1.92 | 1.60E-03 |
| GO:0060089 | F | molecular transducer activity | | 20 | 2.76 | 9.70E-05 |
| GO:0005488 | F | Binding | | 518 | 1.24 | 2.00E-10 |
| GO:0005215 | F | transporter activity | | 80 | 1.64 | 2.10E-05 |
| *Binding activity* |  |  | |  |  |  |
| GO:0046872 | F | metal ion binding | | 173 | 1.36 | 2.50E-05 |
| GO:0015631 | F | tubulin binding | | 18 | 3.59 | 9.70E-06 |
| GO:0003677 | F | DNA binding | | 127 | 1.52 | 3.30E-06 |
| GO:0016168 | F | chlorophyll binding | | 9 | 6.49 | 3.10E-05 |
| GO:0032549 | F | ribonucleoside binding | | 163 | 1.79 | 7.40E-13 |
| GO:0001883 | F | purine nucleoside binding | | 162 | 1.79 | 1.20E-12 |
| GO:0000166 | F | nucleotide binding | | 189 | 1.62 | 5.10E-11 |
| GO:0005524 | F | ATP binding | | 152 | 1.95 | 1.10E-14 |
| *Transferase activity* |  |  | |  |  |  |
| GO:0016740 | F | transferase activity | | 205 | 1.56 | 1.3E-10 |
| **GO term** | **Ontology** | **Description** | | **DEGs (no.)** | **ES^z^** | ***P* value** |
| GO:0016301 | F | kinase activity | | 83 | 1.67 | 8.30E-06 |
| GO:0016773 | F | phosphotransferase activity, alcohol group as acceptor | | 67 | 1.81 | 6.30E-06 |
| GO:0016779 | F | nucleotidyltransferase activity | | 19 | 2.63 | 2.60E-04 |
| GO:0008194 | F | UDP-glycosyltransferase activity | | 24 | 2.54 | 7.10E-05 |
| GO:0016758 | F | transferase activity, transferring hexosyl groups | | 28 | 2.12 | 3.30E-04 |
| *Hydrolase activity* |  |  | |  |  |  |
| GO:0016787 | F | hydrolase activity | | 179 | 1.58 | 1.4E-09 |
| GO:0019203 | F | carbohydrate phosphatase activity | | 6 | 5.98 | 9.80E-04 |
| **Upregulated DEGs** |  |  |  | |  |  |
| Cellular processes |  |  |  | |  |  |
| GO:0009506 | C | Plasmodesma | 54 | | 1.57 | 0.0012 |
| GO:0016020 | C | Membrane | 428 | | 1.31 | 4.30E-11 |
| GO:0071944 | C | cell periphery | 255 | | 1.54 | 9.60E-13 |
| GO:0005622 | C | Intracellular | 847 | | 1.07 | 4.80E-05 |
| Molecular processes |  |  |  | |  |  |
| GO:0005488 | F | Binding | 599 | | 1.29 | 6.70E-17 |
| GO:0003824 | F | catalytic activity | 448 | | 1.29 | 1.50E-10 |
| GO:0004871 | F | signal transducer activity | 32 | | 2.06 | 0.0002 |
| GO:0060089 | F | molecular transducer activity | 22 | | 2.75 | 4.70E-05 |
| *Binding activity* |  |  |  | |  |  |
| GO:0030246 | F | carbohydrate binding | 28 | | 2.41 | 4.10E-05 |
| GO:0005524 | F | ATP binding | 171 | | 1.99 | 4.20E-17 |
| GO:0008270 | F | zinc ion binding | 81 | | 1.63 | 2.20E-05 |
| GO:0005515 | F | protein binding | 204 | | 1.45 | 4.30E-08 |
| *Oxidoreductase activity* |  |  |  | |  |  |
| GO:0016491 | F | oxidoreductase activity | 86 | | 1.45 | 0.00051 |
| GO:0051213 | F | dioxygenase activity | 18 | | 3.48 | 1.40E-05 |
| *Transferase activity* |  |  |  | |  |  |
| GO:0016773 | F | phosphotransferase activity, alcohol group as acceptor | 91 | | 2.22 | 9.80E-12 |
| GO:0016301 | F | kinase activity | 120 | | 2.18 | 9.90E-15 |
| GO:0004675 | F | transmembrane receptor protein serine/threonine kinase activity | 12 | | 3.40 | 0.00044 |
| ^Z^Enrichment score (ES) for individual GO terms was determined by: (DEG number in the GO term/ total DEG number) / (number of DEGs in the genes in the genome for the GO terms / total genes number in the genome); C = cellular processes; F = molecular functions | | | | | | |

| **Supplementary Table S3**. Differentially expressed genes (DEGs) being downregulated relating to photosynthesis, anion transport, and auxin transport in buds of severely symptomatic Huanglongbing (HLB)-affected ‘Hamlin’ sweet orange trees compared to mildly symptomatic trees in February. | | | |
| --- | --- | --- | --- |
| **DEG (*Citrus sinensis*)** | ***Arabidopsis thaliana* ortholog** | **Description** | **Log Fold Change** |
| **GO:0015979 (Photosynthesis)** | |  |  |
| orange1.1g034317m | AT1G29930 | chlorophyll A/B binding protein 1 | -2.02 |
| orange1.1g031166m | AT1G75690 | DnaJ/Hsp40 cysteine-rich domain superfamily protein | -1.68 |
| orange1.1g024782m | AT5G44650 | chloroplast protein-enhancing stress tolerance | -1.31 |
| orange1.1g037299m | AT2G18790 | phytochrome B | -1.22 |
| orange1.1g013014m | AT3G27925 | DegP protease 1 | 1.24 |
| orange1.1g010092m | AT5G24120 | sigma factor E | -1.62 |
| orange1.1g003224m | AT1G54130 | RELA/SPOT homolog 3 | -1.16 |
| orange1.1g013011m | AT4G01050 | thylakoid rhodanese-like | -1.25 |
| orange1.1g022332m | AT1G54780 | thylakoid lumen 18.3 kDa protein | -1.51 |
| orange1.1g022390m | AT3G55800 | sedoheptulose-bisphosphatase | -1.61 |
| orange1.1g013320m | AT1G64810 | *Arabidopsis thaliana* protein of unknown function (DUF794) | -1.22 |
| orange1.1g040346m | AT4G09650 | ATP synthase delta-subunit gene | -1.74 |
| orange1.1g007587m | AT1G16720 | high chlorophyll fluorescence phenotype 173 | -1.35 |
| orange1.1g001056m | AT5G13630 | magnesium-chelatase subunit chlH, chloroplast, putative / Mg-protoporphyrin IX chelatase, putative (CHLH) | -2.09 |
| orange1.1g044971m | AT1G29920 | chlorophyll A/B-binding protein 2 | -2.28 |
| orange1.1g014627m | AT1G17050 | solanesyl diphosphate synthase 2 | -1.56 |
| orange1.1g024579m | AT3G27690 | photosystem II light-harvesting complex gene 2.3 | -1.77 |
| orange1.1g024654m | AT2G34430 | light-harvesting chlorophyll-protein complex II subunit B1 | -2.04 |
| orange1.1g015778m | AT3G03630 | cysteine synthase 26 | -1.28 |
| orange1.1g017014m | AT1G55370 | NDH-dependent cyclic electron flow 5 | -1.54 |
| orange1.1g003913m | AT2G15820 | Endonucleases | -1.25 |
| orange1.1g008143m | AT5G35220 | peptidase M50 family protein | -1.44 |
| orange1.1g029497m | AT3G22840 | chlorophyll A-B binding family protein | -2.17 |
| orange1.1g014877m | AT3G56940 | dicarboxylate diiron protein, putative (Crd1) | -1.35 |
| orange1.1g024422m | AT3G61470 | photosystem I light harvesting complex gene 2 | -1.41 |
| orange1.1g025184m | AT1G77090 | Mog1/PsbP/DUF1795-like photosystem II reaction center PsbP family protein | -1.29 |
| **DEG (*Citrus sinensis*)** | ***Arabidopsis thaliana* ortholog** | **Description** | **Log Fold Change** |
| orange1.1g009449m | AT3G53920 | RNA polymerase sigma-subunit C | -1.80 |
| orange1.1g023899m | AT1G61520 | photosystem I light harvesting complex gene 3 | -1.84 |
| orange1.1g008297m | AT1G68830 | STT7 homolog STN7 | -1.71 |
| orange1.1g015111m | AT3G54050 | high cyclic electron flow 1 | -1.61 |
| orange1.1g011942m | AT1G56190 | phosphoglycerate kinase family protein | -1.34 |
| orange1.1g030898m | AT2G46820 | photosystem I P subunit | -1.42 |
| orange1.1g022529m | AT4G10340 | light harvesting complex of photosystem II 5 | -1.46 |
| orange1.1g032269m | AT1G60950 | 2Fe-2S ferredoxin-like superfamily protein | -1.45 |
| orange1.1g024395m | AT5G54270 | light-harvesting chlorophyll B-binding protein 3 | -2.01 |
| **GO:0006820 (Anion transport)** | |  |  |
| orange1.1g046954m | AT1G67940 | non-intrinsic ABC protein 3 | -1.28 |
| orange1.1g014509m | AT4G17970 | aluminum-activated, malate transporter 12 | -2.33 |
| orange1.1g022448m | AT5G46800 | Mitochondrial substrate carrier family protein | -1.21 |
| orange1.1g012038m | AT3G26570 | phosphate transporter 2;1 | -1.43 |
| orange1.1g014301m | AT5G14570 | high affinity nitrate transporter 2.7 | -1.37 |
| orange1.1g045863m | AT3G19490 | sodium:hydrogen antiporter 1 | -1.26 |
| orange1.1g001847m | AT3G45780 | phototropin 1 | -1.33 |
| orange1.1g012371m | AT1G77690 | like AUX1 3 | -1.64 |
| orange1.1g013096m | AT2G23093 | major facilitator superfamily protein | -1.27 |
| orange1.1g012493m | AT4G24230 | acyl-CoA-binding domain 3 | -1.45 |
| orange1.1g044157m | AT5G19500 | tryptophan/tyrosine permease | -1.40 |
| orange1.1g007736m | AT1G12110 | nitrate transporter 1.1 | -1.83 |
| orange1.1g007159m | AT4G00370 | major facilitator superfamily protein | -1.35 |
| orange1.1g013132m | AT5G46110 | glucose-6-phosphate/phosphate translocator-related | -1.31 |
| orange1.1g004855m | AT2G47160 | HCO3- transporter family | -2.14 |
| orange1.1g000808m | AT1G15520 | pleiotropic drug resistance 12 | -1.27 |
| orange1.1g006373m | AT3G51895 | sulfate transporter 3;1 | -1.64 |
| orange1.1g003778m | AT1G68740 | EXS (ERD1/XPR1/SYG1) family protein | -1.83 |
| **GO:0060918 (Auxin transport)** | |  |  |
| orange1.1g036334m | AT2G26730 | leucine-rich repeat protein kinase family protein | -1.38 |
| orange1.1g002094m | AT5G01890 | leucine-rich receptor-like protein kinase family protein | -1.40 |
| orange1.1g004637m | AT1G14390 | leucine-rich repeat protein kinase family protein | -1.73 |
| orange1.1g002010m | AT1G75820 | leucine-rich receptor-like protein kinase family protein | -1.47 |
| orange1.1g005226m | AT1G67510 | leucine-rich repeat protein kinase family protein | -1.39 |
| orange1.1g003704m | AT4G03390 | STRUBBELIG-receptor family 3 | -1.16 |
| **DEG (*Citrus sinensis*)** | ***Arabidopsis thaliana* ortholog** | **Description** | **Log Fold Change** |
| orange1.1g005693m | AT5G58300 | leucine-rich repeat protein kinase family protein | -1.41 |
| orange1.1g006747m | AT5G58300 | leucine-rich repeat protein kinase family protein | -1.29 |
| orange1.1g001561m | AT5G53890 | phytosylfokine-alpha receptor 2 | -1.48 |
| orange1.1g001566m | AT3G02130 | receptor-like protein kinase 2 | -1.21 |
| orange1.1g036639m | AT2G36570 | leucine-rich repeat protein kinase family protein | -1.36 |
| orange1.1g002717m | AT3G28040 | leucine-rich receptor-like protein kinase family protein | -1.72 |
| orange1.1g004232m | AT5G14210 | leucine-rich repeat protein kinase family protein | -1.60 |
| orange1.1g002721m | AT2G41820 | leucine-rich repeat protein kinase family protein | -1.59 |
| orange1.1g007167m | AT4G23740 | leucine-rich repeat protein kinase family protein | -1.33 |
| orange1.1g006922m | AT4G23740 | leucine-rich repeat protein kinase family protein | -1.40 |
| orange1.1g001867m | AT1G28440 | HAESA-like 1 | -1.67 |
| orange1.1g002121m | AT1G79620 | leucine-rich repeat protein kinase family protein | -1.27 |
| orange1.1g007668m | AT1G31420 | leucine-rich repeat protein kinase family protein | -1.32 |
| orange1.1g048055m | AT1G10850 | leucine-rich repeat protein kinase family protein | -1.51 |
| orange1.1g012745m | AT1G10850 | leucine-rich repeat protein kinase family protein | -1.35 |
| orange1.1g006031m | AT5G43020 | leucine-rich repeat protein kinase family protein | -1.37 |
|  | | | |

| **Supplementary Table S4**. Differentially expressed genes (DEGs) being upregulated relating to response to decreased oxygen levels, osmotic stress, cell death, and defense response incompatible interactions in buds of severely symptomatic Huanglongbing (HLB)-affected ‘Hamlin’ sweet orange trees compared to mildly symptomatic trees in February. | | | |
| --- | --- | --- | --- |
| **DEG (*Citrus sinensis*)** | ***Arabidopsis thaliana* ortholog** | **Description** | **Log Fold Change** |
| **GO:0036293 (response to decreased oxygen levels)** | |  |  |
| orange1.1g047713m | AT1G77120 | alcohol dehydrogenase 1 | 2.16 |
| orange1.1g035170m | AT1G77120 | alcohol dehydrogenase 1 | 3.61 |
| orange1.1g007800m | AT5G54960 | pyruvate decarboxylase 2 | 1.92 |
| orange1.1g011207m | AT3G10040 | sequence-specific DNA binding transcription factors | 5.62 |
| orange1.1g009517m | AT1G72330 | alanine aminotransferase 2 | 1.14 |
| orange1.1g007017m | AT3G20770 | ethylene insensitive 3 family protein | 1.33 |
| orange1.1g044559m | AT4G33070 | Thiamine pyrophosphate-dependent pyruvate decarboxylase family protein | 2.09 |
| orange1.1g016091m | AT2G26150 | heat shock transcription factor A2 | 1.39 |
| orange1.1g038071m | AT2G26150 | heat shock transcription factor A2 | 1.74 |
| orange1.1g026941m | AT4G20380 | LSD1 zinc finger family protein | 1.33 |
| orange1.1g022802m | AT2G26560 | phospholipase A 2A | 1.29 |
| orange1.1g023638m | AT5G15120 | protein of unknown function (DUF1637) | 2.86 |
| **GO:0006970 (response to osmotic stress)** | |  |  |
| orange1.1g041163m | AT5G46050 | peptide transporter 3 | 2.25 |
| orange1.1g007604m | AT5G46050 | peptide transporter 3 | 2.95 |
| orange1.1g007696m | AT5G46050 | peptide transporter 3 | 5.98 |
| orange1.1g011991m | AT2G43850 | Integrin-linked protein kinase family | 1.74 |
| orange1.1g010013m | AT4G23650 | calcium-dependent protein kinase 6 | 1.13 |
| orange1.1g018248m | AT3G62770 | transducin/WD40 repeat-like superfamily protein | 1.52 |
| orange1.1g028954m | AT2G37630 | myb-like HTH transcriptional regulator family protein | 1.18 |
| orange1.1g025162m | AT4G33000 | calcineurin B-like protein 10 | 1.34 |
| orange1.1g039390m | AT5G64750 | Integrase-type DNA-binding superfamily protein | 5.17 |
| orange1.1g012750m | AT5G07440 | glutamate dehydrogenase 2 | 1.60 |
| orange1.1g001061m | AT5G14950 | golgi alpha-mannosidase II | 1.22 |
| **DEG (*Citrus sinensis*)** | ***Arabidopsis thaliana* ortholog** | **Description** | **Log Fold Change** |
| orange1.1g025739m | AT2G36270 | Basic-leucine zipper (bZIP) transcription factor family protein | 1.25 |
| orange1.1g021552m | AT2G26430 | arginine-rich cyclin 1 | 1.14 |
| orange1.1g047713m | AT1G77120 | alcohol dehydrogenase 1 | 2.16 |
| orange1.1g035170m | AT1G77120 | alcohol dehydrogenase 1 | 3.61 |
| orange1.1g026479m | AT2G41130 | basic helix-loop-helix (bHLH) DNA-binding superfamily protein | 1.47 |
| orange1.1g046608m | AT2G39770 | glucose-1-phosphate adenylyltransferase family protein | 1.22 |
| orange1.1g044254m | AT2G37570 | HSP20-like chaperones superfamily protein | 1.31 |
| orange1.1g019628m | AT4G33950 | protein kinase superfamily protein | 1.23 |
| orange1.1g019433m | AT4G33950 | protein kinase superfamily protein | 1.71 |
| orange1.1g011394m | AT3G02360 | 6-phosphogluconate dehydrogenase family protein | 1.22 |
| orange1.1g015012m | AT1G65930 | cytosolic NADP+-dependent isocitrate dehydrogenase | 1.14 |
| orange1.1g039651m | AT5G16510 | alpha-1,4-glucan-protein synthase family protein | 1.30 |
| orange1.1g018800m | AT5G62520 | similar to RCD one 5 | 2.13 |
| orange1.1g009063m | AT1G58200 | MSCS-like 3 | 1.40 |
| orange1.1g042846m | AT3G23250 | myb domain protein 15 | 3.68 |
| orange1.1g036029m | AT4G19810 | glycosyl hydrolase family protein with chitinase insertion domain | 1.89 |
| orange1.1g027915m | AT4G20260 | Plasma-membrane-associated cation-binding protein 1 | 1.17 |
| orange1.1g017231m | AT4G01370 | MAP kinase 4 | 1.40 |
| orange1.1g024492m | AT2G31180 | myb domain protein 14 | 3.13 |
| orange1.1g023728m | AT3G55530 | RING/U-box superfamily protein | 1.18 |
| orange1.1g022980m | AT3G51780 | BCL-2-associated athanogene 4 | 1.48 |
| orange1.1g006816m | AT3G60750 | Transketolase | 3.23 |
| orange1.1g011573m | AT5G40010 | AAA-ATPase 1 | 3.12 |
| orange1.1g021714m | AT5G37850 | pfkB-like carbohydrate kinase family protein | 1.18 |
| orange1.1g004049m | AT1G67580 | protein kinase superfamily protein | 1.26 |
| orange1.1g030727m | AT5G13180 | NAC domain-containing protein 83 | 1.66 |
| orange1.1g018154m | AT4G21440 | MYB-like 102 | 2.51 |
| orange1.1g005406m | AT5G27600 | long-chain acyl-CoA synthetase 7 | 1.21 |
| orange1.1g005468m | AT5G27600 | long-chain acyl-CoA synthetase 7 | 1.31 |
| orange1.1g016322m | AT4G29810 | MAP kinase kinase 2 | 1.27 |
| orange1.1g013391m | AT5G23860 | tubulin beta 8 | 1.17 |
| orange1.1g013071m | AT1G32230 | WWE protein-protein interaction domain protein family | 1.19 |
| orange1.1g045599m | AT5G62530 | aldehyde dehydrogenase 12A1 | 1.30 |
| orange1.1g033293m | AT1G51500 | ABC-2 type transporter family protein | 1.83 |
| orange1.1g011107m | AT1G44170 | aldehyde dehydrogenase 3H1 | 1.16 |
| **DEG (*Citrus sinensis*)** | ***Arabidopsis thaliana* ortholog** | **Description** | **Log Fold Change** |
| orange1.1g027134m | AT4G11600 | glutathione peroxidase 6 | 1.80 |
| **GO:0008219 (cell death)** |  |  |  |
| orange1.1g013592m | AT4G12560 | F-box and associated interaction domains-containing protein | 1.56 |
| orange1.1g014397m | AT3G15010 | RNA-binding (RRM/RBD/RNP motifs) family protein | 1.27 |
| orange1.1g045852m | AT4G26090 | NB-ARC domain-containing disease-resistance protein | 1.52 |
| orange1.1g010895m | AT2G19860 | hexokinase 2 | 1.45 |
| orange1.1g007200m | AT4G33430 | BRI1-associated receptor kinase | 1.30 |
| orange1.1g026941m | AT4G20380 | LSD1 zinc finger family protein | 1.33 |
| orange1.1g007088m | AT5G48380 | BAK1-interacting receptor-like kinase 1 | 2.87 |
| orange1.1g022802m | AT2G26560 | phospholipase A 2A | 1.29 |
| orange1.1g027086m | AT5G51700 | protein binding; zinc ion binding | 1.17 |
| orange1.1g013071m | AT1G32230 | WWE protein-protein interaction domain protein family | 1.19 |
| orange1.1g013270m | AT2G41060 | RNA-binding (RRM/RBD/RNP motifs) family protein | 1.17 |
| orange1.1g027664m | AT4G37980 | elicitor-activated gene 3-1 | 2.43 |
| orange1.1g006910m | AT1G14780 | MAC/Perforin domain-containing protein | 2.03 |
| orange1.1g019887m | AT3G16770 | ethylene-responsive element binding protein | 2.69 |
| orange1.1g048025m | AT4G35350 | xylem cysteine peptidase 1 | 2.62 |
| orange1.1g044857m | AT4G02600 | Seven-transmembrane MLO family protein | 1.85 |
| orange1.1g037847m | AT1G12220 | disease resistance protein (CC-NBS-LRR class) family | 1.56 |
| orange1.1g045137m | AT1G12220 | disease resistance protein (CC-NBS-LRR class) family | 2.09 |
| orange1.1g038165m | AT1G12220 | disease resistance protein (CC-NBS-LRR class) family | 1.33 |
| orange1.1g040898m | AT2G31880 | leucine-rich repeat protein kinase family protein | 1.69 |
| orange1.1g003890m | AT3G46530 | NB-ARC domain-containing disease-resistance protein | 1.83 |
| orange1.1g045150m | AT3G46530 | NB-ARC domain-containing disease-resistance protein | 1.83 |
| orange1.1g011975m | AT3G56860 | UBP1-associated protein 2A | 1.16 |
| orange1.1g018094m | AT4G37990 | elicitor-activated gene 3-2 | 2.45 |
| orange1.1g040495m | AT5G20480 | EF-TU receptor | 2.66 |
| orange1.1g014803m | AT1G08450 | calreticulin 3 | 1.47 |
| orange1.1g046230m | AT5G23670 | long chain base2 | 1.47 |
| orange1.1g002146m | AT1G08720 | protein kinase superfamily protein | 1.25 |
| orange1.1g020187m | AT3G12500 | basic chitinase | 1.87 |
| **GO:0009814 (defense response,**  **incompatible interaction)** | |  |  |
| orange1.1g017231m | AT4G01370 | MAP kinase 4 | 1.40 |
| orange1.1g003890m | AT3G46530 | NB-ARC domain-containing disease-resistance protein | 1.85 |
| orange1.1g045150m | AT3G46530 | NB-ARC domain-containing disease-resistance protein | 1.83 |
| **DEG (*Citrus sinensis*)** | ***Arabidopsis thaliana* ortholog** | **Description** | **Log Fold Change** |
| orange1.1g037847m | AT1G12220 | disease resistance protein (CC-NBS-LRR class) family | 1.56 |
| orange1.1g045137m | AT1G12220 | disease resistance protein (CC-NBS-LRR class) family | 2.09 |
| orange1.1g038165m | AT1G12220 | disease resistance protein (CC-NBS-LRR class) family | 1.33 |
| orange1.1g037010m | AT3G21630 | chitin elicitor receptor kinase 1 | 1.51 |
| orange1.1g006486m | AT4G37870 | phosphoenolpyruvate carboxykinase 1 | 1.49 |
| orange1.1g039202m | AT2G18660 | plant natriuretic peptide A | 2.25 |
| orange1.1g002839m | AT1G55020 | lipoxygenase 1 | 1.51 |
| orange1.1g007366m | AT5G60410 | DNA-binding protein with MIZ/SP-RING zinc finger, PHD-finger, and SAP domain | 1.34 |
| orange1.1g016322m | AT4G29810 | MAP kinase kinase 2 | 1.26 |
| orange1.1g027879m | AT5G38900 | thioredoxin superfamily protein | 1.37 |
| orange1.1g007550m | AT5G45110 | NPR1-like protein 3 | 1.54 |
| orange1.1g013071m | AT1G32230 | WWE protein-protein interaction domain protein family | 1.19 |
| orange1.1g029080m | AT3G25070 | RPM1 interacting protein 4 | 2.20 |
| orange1.1g026941m | AT4G20380 | LSD1 zinc finger family protein | 1.33 |
| orange1.1g020291m | AT3G56400 | WRKY DNA-binding protein 70 | 1.85 |
| orange1.1g038955m | AT4G19660 | NPR1-like protein 4 | 1.48 |
| orange1.1g020187m | AT3G12500 | basic chitinase | 1.85 |
| orange1.1g027086m | AT5G51700 | protein binding; zinc ion binding | 1.17 |
|  | | | |

| **Supplementary Table S5.** Differentially expressed genes (DEGs) relating to transcription factors (TFs), heat shock proteins, resistance (R) genes, pathogenesis-related (PR) proteins, cell wall metabolism, and lipid biosynthesis process in buds of severely symptomatic of Huanglongbing (HLB)-affected ‘Hamlin’ sweet orange trees compared to mildly symptomatic trees in February. | | | |
| --- | --- | --- | --- |
| **DEG (*Citrus sinensis*)** | ***Arabidopsis thaliana* ortholog** | **Description** | **Log Fold Change** |
| Transcription factors (TFs) |  |  |  |
| **WRKY** |  |  |  |
| orange1.1g021896m | AT4G01250 | WRKY family transcription factor | 3.43 |
| orange1.1g020291m | AT3G56400 | WRKY DNA-binding protein 70 | 1.85 |
| orange1.1g019126m | AT4G01250 | WRKY family transcription factor | 2.89 |
| orange1.1g008964m | AT4G04450 | WRKY family transcription factor | 2.46 |
| orange1.1g014629m | AT2G37260 | WRKY family transcription factor family protein | -1.37 |
| orange1.1g020713m | AT2G47260 | WRKY DNA-binding protein 23 | 2.69 |
| orange1.1g015616m | AT5G52830 | WRKY DNA-binding protein 27 | 2.66 |
| orange1.1g025097m | AT1G80840 | WRKY DNA-binding protein 40 | 2.62 |
| **DOF** |  |  |  |
| orange1.1g043493m | AT5G66940 | Dof-type zinc finger DNA-binding family protein | -1.33 |
| orange1.1g021974m | AT5G60200 | TARGET OF MONOPTEROS 6 | -1.33 |
| orange1.1g047290m | AT1G29160 | Dof-type zinc finger DNA-binding family protein | 2.41 |
| orange1.1g011016m | AT5G39660 | cycling DOF factor 2 | -3.29 |
| orange1.1g011752m | AT3G47500 | cycling DOF factor 3 | -3.58 |
| **MYB** |  |  |  |
| orange1.1g042846m | AT3G23250 | myb domain protein 15 | 3.68 |
| orange1.1g003868m | AT1G25360 | pentatricopeptide repeat (PPR) superfamily protein | -1.26 |
| orange1.1g000304m | AT3G52250 | duplicated homeodomain-like superfamily protein | 1.29 |
| orange1.1g025602m | AT1G22640 | myb domain protein 3 | -1.71 |
| orange1.1g024492m | AT2G31180 | myb domain protein 14 | 3.14 |
| orange1.1g047353m | AT4G32730 | homeodomain-like protein | -1.17 |
| orange1.1g018154m | AT4G21440 | MYB-like 102 | 2.51 |
| orange1.1g047854m | AT3G30210 | myb domain protein 121 | -2.16 |
| orange1.1g028954m | AT2G37630 | myb-like HTH transcriptional regulator family protein | 1.18 |
| orange1.1g014308m | AT5G29000 | Homeodomain-like superfamily protein | 1.40 |
| orange1.1g020603m | AT1G25340 | myb domain protein 116 | 2.09 |
| orange1.1g043557m | AT3G28470 | duplicated homeodomain-like superfamily protein | -2.95 |
| **Heat shock proteins** |  |  |  |
| orange1.1g014903m | AT5G22060 | DNAJ homologue 2 | 1.54 |
| orange1.1g042010m | AT3G62190 | chaperone DnaJ-domain superfamily protein | 1.59 |
| **DEG (*Citrus sinensis*)** | ***Arabidopsis thaliana* ortholog** | **Description** | **Log Fold Change** |
| orange1.1g031218m | AT1G07400 | HSP20-like chaperones superfamily protein | -1.31 |
| orange1.1g002068m | AT5G15450 | casein lytic proteinase B3 | -1.22 |
| orange1.1g042521m | AT3G13310 | chaperone DnaJ-domain superfamily protein | 1.61 |
| orange1.1g009246m | AT5G02500 | heat shock cognate protein 70-1 | 2.06 |
| orange1.1g001355m | AT2G29970 | Double Clp-N motif-containing P-loop nucleoside triphosphate hydrolases superfamily protein | -1.38 |
| orange1.1g038071m | AT2G26150 | heat shock transcription factor A2 | 1.74 |
| orange1.1g010831m | AT1G80030 | Molecular chaperone Hsp40/DnaJ family protein | -1.19 |
| orange1.1g008233m | AT2G32120 | heat-shock protein 70T-2 | -1.23 |
| orange1.1g027814m | AT4G13830 | DNAJ-like 20 | 2.55 |
| orange1.1g013732m | AT1G21080 | DNAJ heat shock N-terminal domain-containing protein | 1.37 |
| orange1.1g019573m | AT5G01390 | DNAJ heat shock family protein | 1.28 |
| orange1.1g004296m | AT1G11660 | heat shock protein 70 (Hsp 70) family protein | -1.15 |
| orange1.1g028468m | AT4G25200 | mitochondrion-localized small heat shock protein 23.6 | -1.47 |
| orange1.1g047551m | AT5G49060 | Heat shock protein DnaJ, N-terminal with domain of unknown function (DUF1977) | 1.19 |
| orange1.1g005370m | AT5G52640 | heat shock protein 90.1 | -1.69 |
| **Beta glucanase** |  |  |  |
| orange1.1g045022m | AT2G43670 | Carbohydrate-binding X8 domain superfamily protein | -2.34 |
| orange1.1g039652m | AT3G57270 | beta-1,3-glucanase 1 | 1.99 |
| orange1.1g043682m | AT2G27500 | Glycosyl hydrolase superfamily protein | 2.36 |
| **R genes** |  |  |  |
| orange1.1g034220m | AT2G20142 | Toll-Interleukin-Resistance (TIR) domain family protein | 3.07 |
| orange1.1g000630m | AT1G27170 | transmembrane receptors; ATP binding | 1.27 |
| orange1.1g045613m | AT1G65390 | phloem protein 2 A5 | 2.06 |
| **PR proteins** |  |  |  |
| orange1.1g037371m | AT5G05400 | LRR and NB-ARC domains-containing disease-resistance protein | 1.53 |
| orange1.1g048520m | AT1G50180 | NB-ARC domain-containing disease-resistance protein | 1.59 |
| orange1.1g039821m | AT4G10780 | LRR and NB-ARC domains-containing disease-resistance protein | 1.97 |
| **DEG (*Citrus sinensis*)** | ***Arabidopsis thaliana* ortholog** | **Description** | **Log Fold Change** |
| orange1.1g029746m | AT1G58170 | disease resistance-responsive (d family protein | -1.36 |
| orange1.1g020066m | AT3G50950 | HOPZ-ACTIVATED RESISTANCE 1 | 1.45 |
| orange1.1g002167m | AT5G27060 | Receptor-like protein 53 | 1.79 |
| orange1.1g038419m | AT1G61100 | disease resistance protein (TIR class), putative | -1.30 |
| orange1.1g031657m | AT5G35450 | disease resistance protein (CC-NBS-LRR class) family | 1.28 |
| orange1.1g019584m | AT1G47890 | Receptor-like protein 7 | 3.29 |
| orange1.1g036637m | AT1G61190 | LRR and NB-ARC domains-containing disease-resistance protein | 1.60 |
| orange1.1g041071m | AT1G69550 | disease resistance protein (TIR-NBS-LRR class) | 2.62 |
| orange1.1g042811m | AT1G12290 | disease resistance protein (CC-NBS-LRR class) family | 1.34 |
| orange1.1g046642m | AT2G32660 | Receptor-like protein 22 | 2.23 |
| orange1.1g008628m | AT1G71400 | Receptor-like protein 12 | 3.36 |
| orange1.1g022670m | AT1G15890 | disease resistance protein (CC-NBS-LRR class) family | 2.22 |
| orange1.1g041476m | AT5G63020 | disease resistance protein (CC-NBS-LRR class) family | 2.09 |
| orange1.1g048831m | AT5G17680 | disease resistance protein (TIR-NBS-LRR class), putative | 3.85 |
| orange1.1g041683m | AT2G34930 | disease resistance family protein / LRR family protein | 4.08 |
| orange1.1g012027m | AT5G36930 | disease resistance protein (TIR-NBS-LRR class) family | 9.64 |
| orange1.1g041797m | AT2G39430 | disease resistance-responsive (dirigent-like protein) family protein | -1.95 |
| orange1.1g037483m | AT4G12010 | disease resistance protein (TIR-NBS-LRR class) family | 3.01 |
| orange1.1g026255m | AT5G40170 | Receptor-like protein 54 | 2.99 |
| orange1.1g043908m | AT2G17050 | disease resistance protein (TIR-NBS-LRR class), putative | -1.44 |
| orange1.1g025557m | AT4G19470 | leucine-rich repeat (LRR) family protein | 2.16 |
| orange1.1g040303m | AT4G27220 | NB-ARC domain-containing disease-resistance protein | 1.66 |
| orange1.1g044398m | AT1G55210 | disease resistance-responsive (dirigent-like protein) family protein | 2.71 |
| orange1.1g007039m | AT4G27190 | NB-ARC domain-containing disease-resistance protein | 2.73 |
| orange1.1g048827m | AT5G23400 | leucine-rich repeat (LRR) family protein | -1.49 |
| orange1.1g046764m | AT3G14460 | LRR and NB-ARC domains-containing disease-resistance protein | 6.54 |
| orange1.1g043910m | AT5G45200 | disease resistance protein (TIR-NBS-LRR class) family | 3.09 |
| orange1.1g007268m | AT1G61180 | LRR and NB-ARC domains-containing disease-resistance protein | 2.00 |
| orange1.1g002044m | AT3G14470 | NB-ARC domain-containing disease-resistance protein | 4.53 |
| orange1.1g040238m | AT4G13810 | Receptor-like protein 47 | 2.36 |
| orange1.1g002435m | AT3G11080 | Receptor-like protein 35 | 2.43 |
| **DEG (*Citrus sinensis*)** | ***Arabidopsis thaliana* ortholog** | **Description** | **Log Fold Change** |
| **Cell wall metabolism** |  |  |  |
| orange1.1g046608m | AT2G39770 | glucose-1-phosphate adenylyltransferase family protein | 1.22 |
| orange1.1g001402m | AT1G01220 | L-fucokinase/GDP-L-fucose pyrophosphorylase | -1.13 |
| orange1.1g019795m | AT1G12780 | UDP-D-glucose/UDP-D-galactose 4-epimerase 1 | 1.64 |
| orange1.1g026752m | AT4G23920 | UDP-D-glucose/UDP-D-galactose 4-epimerase 2 | 1.21 |
| orange1.1g014011m | AT3G53520 | UDP-glucuronic acid decarboxylase 1 | -1.16 |
| orange1.1g042406m | AT4G00110 | UDP-D-glucuronate 4-epimerase 3 | 1.49 |
| orange1.1g005818m | AT1G78570 | rhamnose biosynthesis 1 | -1.21 |
| orange1.1g019775m | AT3G54690 | sugar isomerase (SIS) family protein | -1.21 |
| orange1.1g041333m | AT5G03760 | nucleotide-diphospho-sugar transferases superfamily protein | -1.66 |
| orange1.1g040333m | AT5G22740 | cellulose synthase-like A02 | -1.43 |
| orange1.1g001373m | AT2G21770 | cellulose synthase A9 | -1.29 |
| orange1.1g043222m | AT4G24000 | cellulose synthase-like G2 | -1.55 |
| orange1.1g041635m | AT4G24010 | cellulose synthase-like G1 | -1.67 |
| orange1.1g008917m | AT2G03220 | fucosyltransferase 1 | 1.82 |
| orange1.1g005683m | AT3G61130 | galacturonosyltransferase 1 | -1.22 |
| orange1.1g037512m | AT5G03170 | Fasciclin -like arabinogalactan-protein 11 | -4.66 |
| orange1.1g035993m | AT2G47930 | arabinogalactan protein 26 | -1.97 |
| orange1.1g041580m | AT4G37450 | arabinogalactan protein 18 | -1.42 |
| orange1.1g024965m | AT2G04780 | Fasciclin -like arabinogalactan 7 | -1.35 |
| orange1.1g044183m | AT3G46550 | Fasciclin-like arabinogalactan family protein | -1.16 |
| orange1.1g019153m | AT5G06390 | Fasciclin -like arabinogalactan protein 17 precursor | -1.44 |
| orange1.1g013841m | AT4G12730 | Fasciclin-like arabinogalactan 2 | -1.71 |
| orange1.1g039087m | AT4G29240 | leucine-rich repeat (LRR) family protein | -1.52 |
| orange1.1g044868m | AT2G42800 | Receptor-like protein 29 | -1.83 |
| orange1.1g039651m | AT5G16510 | alpha-1,4-glucan-protein synthase family protein | 1.30 |
| orange1.1g009690m | AT2G32990 | glycosyl hydrolase 9B8 | -1.45 |
| orange1.1g047621m | AT5G09730 | beta-xylosidase 3 | -1.96 |
| orange1.1g039255m | AT4G30270 | xyloglucan endotransglucosylase/hydrolase 24 | 7.41 |
|  |  |  |  |
| **DEG (*Citrus sinensis*)** | ***Arabidopsis thaliana* ortholog** | **Description** | **Log Fold Change** |
| orange1.1g040748m | AT1G02460 | pectin lyase-like superfamily protein | -1.42 |
| orange1.1g011204m | AT5G04310 | pectin lyase-like superfamily protein | -1.40 |
| orange1.1g012057m | AT1G48100 | pectin lyase-like superfamily protein | -1.84 |
| orange1.1g011659m | AT4G23500 | pectin lyase-like superfamily protein | -1.37 |
| orange1.1g012308m | AT3G62110 | pectin lyase-like superfamily protein | -1.51 |
| orange1.1g038762m | AT3G57790 | pectin lyase-like superfamily protein | 1.34 |
| orange1.1g039202m | AT2G18660 | plant natriuretic peptide A | 2.25 |
| orange1.1g025740m | AT1G69530 | expansin A1 | -2.11 |
| orange1.1g009085m | AT5G09760 | plant invertase/pectin methylesterase inhibitor superfamily | -1.39 |
| orange1.1g019696m | AT5G19730 | pectin lyase-like superfamily protein | -1.61 |
| orange1.1g018173m | AT3G14310 | pectin methylesterase 3 | -1.16 |
| orange1.1g044734m | AT2G26440 | plant invertase/pectin methylesterase inhibitor superfamily | -1.39 |
| orange1.1g031901m | AT4G33220 | pectin methylesterase 44 | 1.19 |
| **Lignin biosynthesis** |  |  |  |
| orange1.1g012770m | AT2G30490 | cinnamate-4-hydroxylase | 5.69 |
| orange1.1g038977m | AT1G65060 | 4-coumarate: CoA ligase 3 | -1.60 |
| orange1.1g044836m | AT4G34050 | S-adenosyl-L-methionine-dependent methyltransferases superfamily protein | -1.82 |
| orange1.1g042491m | AT5G54160 | O-methyltransferase 1 | 2.79 |
| orange1.1g018094m | AT4G37990 | elicitor-activated gene 3-2 | 2.45 |
| orange1.1g027664m | AT4G37980 | elicitor-activated gene 3-1 | 2.42 |
| orange1.1g018158m | AT4G39330 | cinnamyl alcohol dehydrogenase 9 | -1.64 |
| orange1.1g018382m | AT3G19450 | GroES-like zinc-binding alcohol dehydrogenase family protein | 1.48 |

| **Supplementary Table S6.** Differentially expressed genes (DEGs) relating to redox, antioxidants properties, fatty acid beta-oxidation, autophagy, and hormones metabolism in buds of severely symptomatic of Huanglongbing (HLB)-affected ‘Hamlin’ sweet orange trees compared to mildly symptomatic trees in February. | | | | |
| --- | --- | --- | --- | --- |
| **ROS scavenging mechanism** | **DEGs**  **(*Citrus sinensis*)** | ***Arabidopsis thaliana* ortholog** | **Description** | **Log Fold Change** |
| Redox thioredoxin |  |  |  |  |
|  | orange1.1g010804m | AT1G21750 | redox thioredoxin (PDI-like 1-1) | -1.28 |
|  | orange1.1g030165m | AT4G03520 | redox thioredoxin (thioredoxin superfamily protein) | -1.87 |
|  | orange1.1g033947m | AT5G16400 | redox thioredoxin (thioredoxin F2) | -1.37 |
|  | orange1.1g018973m | AT1G76080 | redox thioredoxin (chloroplastic drought-induced stress protein of 32 kD) | -1.51 |
|  | orange1.1g033251m | AT3G51030 | redox thioredoxin (thioredoxin H-type 1) | 2.16 |
| Redox glutaredoxins |  |  |  |  |
|  | orange1.1g044679m | AT1G64500 | redox glutaredoxins (glutaredoxin family protein) | -1.95 |
|  | orange1.1g027825m | AT1G64500 | redox glutaredoxins (glutaredoxin family protein) | -1.84 |
|  | orange1.1g034205m | AT2G30540 | redox glutaredoxins (thioredoxin superfamily protein) | 2.13 |
|  | orange1.1g034075m | AT5G18600 | redox glutaredoxins (thioredoxin superfamily protein) | 1.83 |
| Peroxidases |  |  |  |  |
|  | orange1.1g019585m | AT5G17820 | peroxidase superfamily protein | 3.56 |
|  | orange1.1g036989m | AT2G34060 | peroxidase superfamily protein | -1.78 |
| Ascorbate peroxidase |  |  |  |  |
|  | orange1.1g024615m | AT1G07890 | ascorbate peroxidase 1 | -1.17 |
| Glutathione peroxidase | |  |  |  |
|  | orange1.1g030881m | AT1G63460 | glutathione peroxidase 8 | 1.22 |
|  | orange1.1g027134m | AT4G11600 | glutathione peroxidase 6 | 1.80 |
| Glutathione S transferase | |  |  |  |
|  | orange1.1g026781m | AT2G02390 | glutathione S-transferase zeta 1 | 1.26 |
|  | orange1.1g041800m | AT2G29420 | glutathione S-transferase tau 7 | 1.49 |
|  | orange1.1g027333m | AT2G29420 | glutathione S-transferase tau 7 | 2.75 |
|  | orange1.1g027524m | AT2G29420 | glutathione S-transferase tau 7 | 2.77 |
|  | orange1.1g036834m | AT3G09270 | glutathione S-transferase TAU 8 | 2.23 |
|  | orange1.1g031084m | AT2G30860 | glutathione S-transferase PHI 9 | 2.39 |
|  | orange1.1g037448m | AT1G10360 | glutathione S-transferase TAU 18 | 2.41 |
|  | orange1.1g047906m | AT2G29420 | glutathione S-transferase tau 7 | 1.79 |
|  | orange1.1g033818m | AT3G09270 | glutathione S-transferase TAU 8 | 1.92 |
|  | orange1.1g040684m | AT5G42150 | glutathione S-transferase family protein | 1.22 |
|  | orange1.1g023929m | AT2G29420 | glutathione S-transferase tau 7 | 1.63 |
| **ROS scavenging mechanism** | **DEGs**  **(*Citrus sinensis*)** | ***Arabidopsis thaliana* ortholog** | **Description** | **Log Fold Change** |
| Proline |  |  |  |  |
|  | orange1.1g019781m | AT2G32600 | hydroxyproline-rich glycoprotein family protein | 1.11 |
|  | orange1.1g022067m | AT2G32840 | proline-rich family protein | 1.28 |
|  | orange1.1g007230m | AT5G38600 | proline-rich spliceosome-associated (PSP) family protein | 1.36 |
|  | orange1.1g020588m | AT2G39890 | proline transporter 1 | 1.80 |
| Oxidative stress (Fatty acid beta-oxidation) | |  |  |  |
|  | orange1.1g026137m | AT4G14440 | 3-hydroxyacyl-CoA dehydratase 1 | 1.47 |
|  | orange1.1g041046m | AT4G14440 | 3-hydroxyacyl-CoA dehydratase 1 | 1.43 |
|  | orange1.1g026127m | AT4G14430 | indole-3-butyric acid response 10 | 1.31 |
|  | orange1.1g015820m | AT4G31810 | ATP-dependent caseinolytic (Clp) protease/crotonase family protein | 1.19 |
|  | orange1.1g010304m | AT3G58750 | citrate synthase 2 | 1.19 |
|  | orange1.1g022503m | AT3G51840 | acyl-CoA oxidase 4 | 2.73 |
|  | orange1.1g012506m | AT2G33150 | peroxisomal 3-ketoacyl-CoA thiolase 3 | 1.30 |
|  | orange1.1g042147m | AT4G16760 | acyl-CoA oxidase 1 | 1.82 |
|  | orange1.1g007504m | AT4G16760 | acyl-CoA oxidase 1 | 1.93 |
|  | orange1.1g022548m | AT3G07560 | peroxin 13 | 1.39 |
|  | orange1.1g022421m | AT3G07560 | peroxin 13 | 1.36 |
| **Hormones** |  |  |  |  |
| Jasmonic acid | orange1.1g002839m | AT1G55020 | lipoxygenase 1 | 1.51 |
|  | orange1.1g039030m | AT5G42650 | allene oxide synthase | 2.87 |
|  | orange1.1g015862m | AT1G76690 | 12-oxophytodienoate reductase 2 | 1.80 |
|  | orange1.1g046574m | AT1G76680 | 12-oxophytodienoate reductase 1 | 2.19 |
| Abscisic acid |  |  |  |  |
|  | orange1.1g043931m | AT5G08350 | GRAM domain-containing protein / ABA-responsive protein-related | 1.85 |
|  | orange1.1g029408m | AT2G22475 | GRAM domain family protein | 1.26 |
| Brassinosteroids |  |  |  |  |
|  | orange1.1g038451m | AT5G08130 | basic helix-loop-helix (bHLH) DNA-binding superfamily protein | -1.73 |
|  | orange1.1g019700m | AT2G03760 | sulphotransferase 12 | -1.42 |
|  | orange1.1g020318m | AT1G78700 | BES1/BZR1 homolog 4 | -1.26 |
| Brassinosteroids | orange1.1g043046m | AT3G02580 | sterol 1 | -1.21 |
|  | orange1.1g007200m | AT4G33430 | BRI1-associated receptor kinase | 1.30 |
|  | orange1.1g030807m | AT3G61460 | brassinosteroids-responsive RING-H2 | 1.72 |
|  | orange1.1g030257m | AT2G01950 | BRI1-like 2 | 2.49 |
| **ROS scavenging mechanism** | **DEGs**  **(*Citrus sinensis*)** | ***Arabidopsis thaliana* ortholog** | **Description** | **Log Fold Change** |
| Salicylic acid |  |  |  |  |
|  | orange1.1g045960m | AT5G66430 | S-adenosyl-L-methionine-dependent methyltransferases superfamily protein | 1.67 |
|  | orange1.1g016644m | AT5G55250 | IAA carboxyl methyltransferase 1 | -1.65 |
| Ethylene |  |  |  |  |
|  | orange1.1g014328m | AT4G31980 | peptidase activity | -5.32 |
|  | orange1.1g036196m | AT3G19500 | basic helix-loop-helix (bHLH) DNA-binding superfamily protein | -1.79 |
|  | orange1.1g032263m | AT2G44840 | ethylene-responsive element binding factor 13 | -1.75 |
|  | orange1.1g035657m | AT1G27660 | basic helix-loop-helix (bHLH) DNA-binding superfamily protein | -1.68 |
|  | orange1.1g039774m | AT1G03400 | 2-oxoglutarate (2OG) and Fe (II)-dependent oxygenase superfamily protein | 1.36 |
|  | orange1.1g046659m | AT2G30830 | 2-oxoglutarate (2OG) and Fe (II)-dependent oxygenase superfamily protein | 1.39 |
|  | orange1.1g015846m | AT4G17500 | ethylene responsive element binding factor 1 | 2.42 |
|  | orange1.1g043923m | AT4G37770 | 1-amino-cyclopropane-1-carboxylate synthase 8 | 2.91 |
|  | orange1.1g045963m | AT4G34410 | redox responsive transcription factor 1 | 4.86 |
| Auxins |  |  |  |  |
|  | orange1.1g036242m | AT5G47530 | auxin-responsive family protein | -2.29 |
|  | orange1.1g012371m | AT1G77690 | like AUX1 3 | -1.64 |
|  | orange1.1g007568m | AT4G03400 | Auxin-responsive GH3 family protein | -1.61 |
|  | orange1.1g019368m | AT1G60710 | NAD(P)-linked oxidoreductase superfamily protein | -1.59 |
|  | orange1.1g047801m | AT3G25290 | auxin-responsive family protein | -1.51 |
|  | orange1.1g041574m | AT1G77110 | auxin efflux carrier family protein | -1.48 |
|  | orange1.1g001093m | AT2G34680 | outer arm dynein light chain 1 protein | -1.34 |
|  | orange1.1g010411m | AT1G14020 | O-fucosyltransferase family protein | -1.18 |
|  | orange1.1g008073m | AT1G48660 | Auxin-responsive GH3 family protein | 1.31 |
|  | orange1.1g020083m | AT1G60690 | NAD(P)-linked oxidoreductase superfamily protein | 1.37 |
|  | orange1.1g036854m | AT4G22620 | SAUR-like auxin-responsive protein family | 1.95 |
|  | orange1.1g034194m | AT5G53590 | SAUR-like auxin-responsive protein family | 2.22 |
|  | orange1.1g042896m | AT1G24100 | UDP-glucosyl transferase 74B1 | 2.77 |
|  | orange1.1g025251m | AT4G27450 | Aluminium-induced protein with YGL and LRDR motifs | 2.85 |
|  | orange1.1g032663m | AT1G72430 | SAUR-like auxin-responsive protein family | 2.97 |
